# Accelerating GPCR Drug Discovery With Conformation-Stabilizing VHHs

**DOI:** 10.3389/fmolb.2022.863099

**Published:** 2022-05-23

**Authors:** Toon Laeremans, Zara A. Sands, Pieter Claes, Ann De Blieck, Stephane De Cesco, Sarah Triest, Andreas Busch, David Felix, Abhinav Kumar, Veli-Pekka Jaakola, Christel Menet

**Affiliations:** Confo Therapeutics NV, Zwijnaarde, Belgium

**Keywords:** active state, biosensor, ConfoBody, conformation, conformer, GPCR, SBDD, VHH

## Abstract

The human genome encodes 850 G protein-coupled receptors (GPCRs), half of which are considered potential drug targets. GPCRs transduce extracellular stimuli into a plethora of vital physiological processes. Consequently, GPCRs are an attractive drug target class. This is underlined by the fact that approximately 40% of marketed drugs modulate GPCRs. Intriguingly 60% of non-olfactory GPCRs have no drugs or candidates in clinical development, highlighting the continued potential of GPCRs as drug targets. The discovery of small molecules targeting these GPCRs by conventional high throughput screening (HTS) campaigns is challenging. Although the definition of success varies per company, the success rate of HTS for GPCRs is low compared to other target families (Fujioka and Omori, 2012; Dragovich et al., 2022). Beyond this, GPCR structure determination can be difficult, which often precludes the application of structure-based drug design approaches to arising HTS hits. GPCR structural studies entail the resource-demanding purification of native receptors, which can be challenging as they are inherently unstable when extracted from the lipid matrix. Moreover, GPCRs are flexible molecules that adopt distinct conformations, some of which need to be stabilized if they are to be structurally resolved. The complexity of targeting distinct therapeutically relevant GPCR conformations during the early discovery stages contributes to the high attrition rates for GPCR drug discovery programs. Multiple strategies have been explored in an attempt to stabilize GPCRs in distinct conformations to better understand their pharmacology. This review will focus on the use of camelid-derived immunoglobulin single variable domains (VHHs) that stabilize disease-relevant pharmacological states (termed ConfoBodies by the authors) of GPCRs, as well as GPCR:signal transducer complexes, to accelerate drug discovery. These VHHs are powerful tools for supporting in vitro screening, deconvolution of complex GPCR pharmacology, and structural biology purposes. In order to demonstrate the potential impact of ConfoBodies on translational research, examples are presented of their role in active state screening campaigns and structure-informed rational design to identify *de novo* chemical space and, subsequently, how such matter can be elaborated into more potent and selective drug candidates with intended pharmacology.

## Introduction

G protein-coupled receptors (GPCRs) represent a major therapeutic target class as they play a key role in many (patho-) physiological processes. GPCRs are divided into six classes based on amino acid sequence similarities, but only four of the classes (A, B, C, and F) are found in humans. GPCRs respond to a wide variety of signals that range in size from photons to proteins (Foord et al., 2005). GPCRs continue to be regarded as one of the most tractable classes of drug targets and are targeted by 30%–40% of current drugs (Hauser et al., 2017), with annual sales of GPCR-targeting drugs in 2018 accounting for >114 billion USD. In 2019, 5 out of 20 first-in-class approved therapeutic agents targeted GPCRs. Despite this high number of GPCR targeted drugs, only a small portion (∼110) of the human GPCRome (consisting of approximately 850 GPCRs) has been successfully drugged, and obtaining highly potent and selective small molecules remains a challenge for the remainder.

All GPCRs contain seven membrane-spanning α-helices and are conformationally highly dynamic. Upon stimulation, GPCRs undergo a conformational switch that enables their coupling with cytosolic signal-transducing proteins such as G proteins, β-arrestin, and other effector proteins. The ligand-dictated recruitment of cytosolic signal transducers results in the activation of signaling pathways that eventually lead to a particular biological response. Upon activation, the intracellular ends of transmembrane (TM) helices TM5 and TM6 move outward to form an allosteric pocket in the receptor where the C-terminus of the G protein α-subunit binds, stabilizing the active state of the receptor (Rosenbaum et al., 2009). The conformational complexity of the GPCR allows drugs with different profiles to act *via* multiple pathways. The ligand-dependent activation of certain pathways over others, which can lead to a “functionally selective” response, is a phenomenon known as biased agonism (Smith et al., 2018).

Despite the long history of the development of drugs targeting GPCRs, the challenges associated with their discovery are numerous. Most recent GPCR drugs have arisen from hits initially identified *via* high through-put screening campaigns against large compound collections. However, there remain a high number of “undrugged” GPCRs with high therapeutic potential. This, in part, can be attributed to the fact that compound collections used for screening are not diverse enough and ultimately lack the features required to modulate the intended GPCR target. Further, the druggable pockets of GPCRs may adopt an ensemble of conformations that are transiently sampled. Consequently, identifying GPCR-specific chemical starting points with desired pharmacology remains challenging. These challenges have recently been approached using structure-based drug design (SBDD) and fragment screening, fueled by the progress in the GPCR structural biology field over the last 15 years. However, while the inactive state conformation can be obtained by X-ray crystallography in the presence of only an antagonist, the fully active state protein structure of the GPCR can only be obtained in the presence of an agonist in addition to a molecular chaperone that cooperatively stabilizes the active state receptor. Similarly, fragment screening can be performed using biophysical approaches to identify antagonists successfully. However, the discovery of agonists requires the use of conformation-specific tools (Congreve et al., 2017). The preparation of purified GPCRs for such structure and biophysical studies requires significant quantities of purified protein, which is not only resource-demanding but challenging, as GPCRs are unstable when extracted from their membrane context.

VHHs (synonyms in the scientific literature include nanobodies or sdAbs) are small proteins (circa 12–15 kDa) comprised of single variable fragments of heavy-chain-only antibodies found in members of the Camelidae family. Although VHHs that interact with linear epitopes have been reported (Cheloha et al., 2020b), contrary to antigen interaction with conventional antibodies, VHHs often contain a long hypervariable complementary determining region 3 (CDR3) segment, which enables binding to discontinuous cryptic epitopes, cavities, or clefts on the surface of proteins (Lauwereys et al., 1998; Stijlemans et al., 2004; De Genst et al., 2006). While preferably interacting with discontinuous epitopes, VHHs have similar antigen affinity ranges compared to conventional antibodies, are biochemically more stable molecules (less prone to aggregation upon recombinant expression hence easier to produce and purify), and are compatible with robust recombinant display techniques, including phage, yeast, bacterial, and ribosome display (Muyldermans 2013). For these reasons, VHHs are ideal tools to selectively stabilize desired conformational states of conformationally complex (membrane) proteins such as GPCRs. In this review, the authors will further refer to such conformation-stabilizing immunoglobulin single variable domains as ConfoBodies^1^ (Cbs).

In order to improve the understanding of GPCR pharmacology, multiple strategies have explored molecular techniques to stabilize GPCRs in their distinct conformations: thermostabilized GPCRs; heterotrimeric mini-G proteins or G protein-peptide derivatives; and conformer-stabilizing immunoglobulin fragments or synthetic aptamer scaffolds. The scope of this review will be on Cbs that stabilize GPCR conformations by interacting with the cytosolic domain of a receptor (either by direct interaction with the intracellular epitopes of a GPCR or by indirectly interacting with a signaling transducer coupled to a receptor). We will describe how these Cbs are identified and how these tools will open up new avenues for GPCR drug discovery. By their ability to stabilize inactive or active GPCR conformers, these Cbs have been extensively used as key reagents to determine active and inactive state GPCR protein structures. These tools have also led to new approaches for drug discovery by enabling GPCR agonist fragment screening followed by SBDD. Finally, these approaches have led to the development of VHHs as biosensors to investigate GPCR signaling (Irannejad et al., 2013; Staus et al., 2014; Staus et al., 2016; Stoeber et al., 2018). For uniform GPCR annotation in this review, the UniProt gene name or one of the synonyms is used (www.uniprot.org).

## Discovery of GPCR Active and Inactive State-Stabilizing ConfoBodies

**FIGURE 1:**
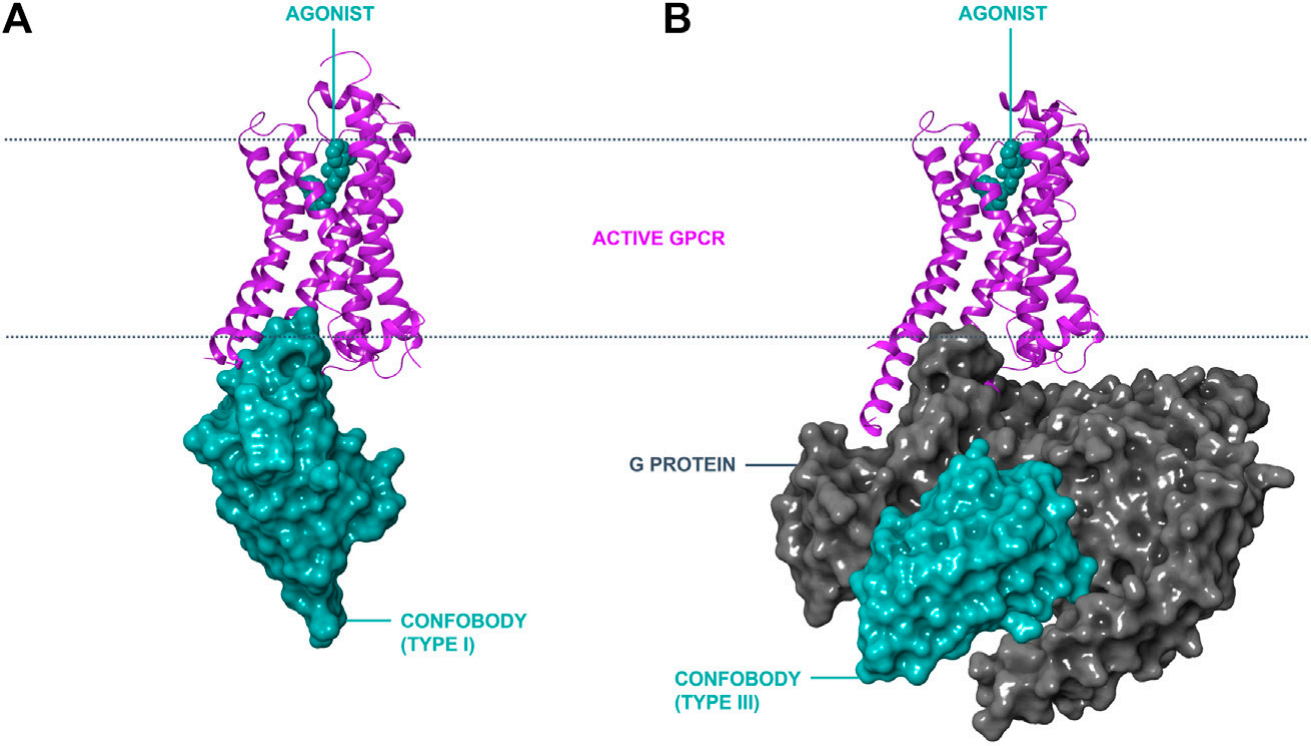
Modes of action of conformation**-**stabilizing VHHs (ConfoBodies; Cbs). Cbs can stabilize disease-relevant conformational states of GPCRs as exemplified by the active state ADRB2:Nb80 structure (PDB code 3P0G) depicted in panel **(A)**. A similar mode of interaction to a GPCR by Type II Cbs (stabilizing the inactive state conformer) is possible by protruding in the cytosolic receptor pocket (not depicted). Cbs can also stabilize protein complexes such as active GPCR:G protein complexes, as illustrated by the ADRB2:Gs:Nb35 structure (PDB code 3SN6) in panel **(B)**. ADRB2 is represented in magenta ribbon format; Cbs are depicted in cyan surface format; the Gs complex is shown in grey surface format; and the small molecule orthosteric agonist BI-167107 is shown in cyan CPK format the lipid bilayer is delineated with a dotted line.

Inactive state conformer-stabilizing conventional antibody fragments have been reported against GPCRs (e.g., Fab2838 to A2A; Hino et al., 2012). However, Cbs are the only antibody-derived scaffolds reported thus far that stabilize active GPCR conformers. Three different types of Cbs are described in this review (Table 1). Type I Cbs are G protein-mimicking VHHs that directly interact with the intracellular loops of a GPCR and stabilize the active conformational state (Figure 1, panel A). Type II Cbs are negative allosteric modulator (NAM) VHHs that directly interact with the intracellular loops of a GPCR and stabilize the inactive conformer. Type III Cbs are transducer-stabilizing VHHs. These indirectly stabilize a GPCR in an active conformer by interacting with a downstream signaling transducer protein, such as a G protein, bound to the GPCR (Figure 1, panel B). An overview of all reported Type I–III ConfoBodies is provided in Supplementary Table S1.

**TABLE 1:** Conformer-stabilizing VHH (ConfoBody) classification used in this review article.

Type of conformer-stabilizing VHH	Mode of action	Conformer specificity	Epitope	Example and reference
I.	G protein mimetic	Active state	Intracellular	Nb80 ADRB2; Rasmussen et al. (2011a)
II.	Negative allosteric modulator (NAM)	Inactive state	Intracellular	Nb60 ADRB2; Staus et al. (2014)
III.	Transducer stabilizing	Active state	Intracellular *via* transducer	Nb35 G protein; Rasmussen et al. (2011b)

G protein-mimicking Type I Cbs have been reported for ADRB1/2, ACM2, OPRM, OPRK, US28, AGTR1, AA2AR, and SMO. Inactive state-stabilizing Type II Cbs Nb60, Nb6, and Nanobody6 have been reported against ADRB2, OPRK, and succinate receptor SUCR1, respectively. Transducer-stabilizing Type III Cbs have been identified for Gs, Gq, and β-arrestin.

Following a joint effort between the Kobilka and Steyaert labs, Rasmussen et al. (2011a) reported the first G protein-mimicking Cb (Nb80) selective for the active state conformer of ADRB2. This pioneering work described the use of Nb80 to determine the ADRB2 active state crystal structure. Protruding into the receptor’s cytosolic cavity, Nb80 was found to stabilize the agonist-occupied ADRB2 signaling conformer (Figure 1, panel A). A second landmark paper by Kobilka’s group described the active state structure of ADRB2 in complex with the Gs G protein (Rasmussen et al., 2011b). The conformer-stabilizing, Type III transducer-stabilizing ConfoBody Nb35 was utilized as a molecular chaperone (Supplementary Table S1) and was key to obtaining this molecular snapshot of the G protein signal transducer bound to the agonist-occupied ADRB2. The epitope of Nb35 is shaped by the interface of the Gαs and the Gβ subunit of the G protein when bound to the agonist-occupied receptor. This study confirms that the ADRB2 active state structure obtained with Nb35 is nearly identical to the structure using Nb80 as a chaperone, confirming Nb80 as an excellent G protein mimetic.

In addition, large panels of VHHs that target extracellular epitopes of native class A (CXCR4, CXCR2, ACKR3, CX3C1, US28, CML1, APJ, OX2R, AGTR2, and OPRM), B (GCGR, GLP1R, PTH1R, and VPAC1), and C (mGluR2, CaSR, mGluR4, and mGluR5) GPCRs have been described (Jähnichen et al., 2010; Huang et al., 2015; Peyrassol et al., 2016; Peyrassol et al., 2018; Van Hout et al., 2018; Koehl et al., 2019; Cheloha et al., 2020a; Low et al., 2020; Pan et al., 2020; Ren et al., 2020; De Groof et al., 2021; Chen et al., 2021; Haubrich et al., 2021 and references in Heukers et al., 2019). While most of these VHHs that target extracellular epitopes of GPCRs block receptor signaling or are not demonstrated to interfere with receptor signal transduction, some of these extracellular binders are reported to ortho- or allosterically activate receptor signaling, thus inherently stabilizing active GPCR conformers (Ma et al., 2020; Ren et al., 2020; Hong et al., 2021; Scholler et al., 2017). VHHs that target extracellular epitopes of GPCRs are out of the scope of this review. Instead, we will focus on ConfoBodies that stabilize GPCR conformers *via* the cytosolic pocket, for which conformer stabilization is unambiguously demonstrated.

### Recombinant Display Technologies to Mine Large VHH Repertoires for ConfoBodies

**FIGURE 2:**
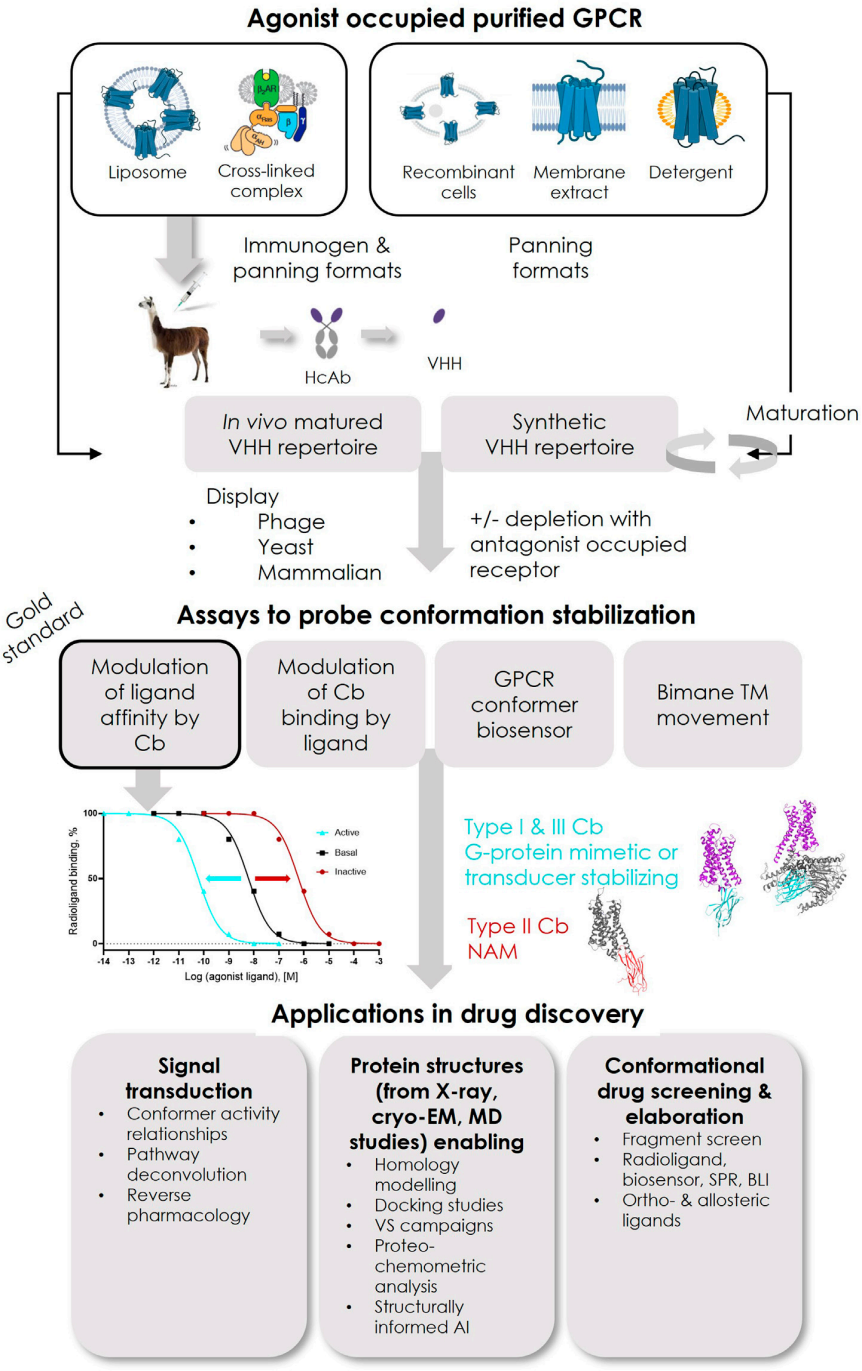
Overview of reported successful Type I and Type III ConfoBody discovery strategies and applications of ConfoBodies in GPCR drug discovery. Immunogens that so far contributed to the identification of active state ConfoBodies are purified protein reconstituted in liposomes or a cross-linked complex. GPCR configurations that have been successfully applied as panning formats in combination with the different display methods (phage, yeast, or mammalian display) are purified protein reconstituted in liposomes or as detergent micelle or a cross-linked complex with a downstream transducer or mammalian cells overexpressing recombinant receptor (or derived membrane extracts). The combinations of immunogen and panning formats that delivered Cbs can be extracted from Supplementary Table S1. Abbreviations: artificial intelligence (AI), biolayer interferometry (BLI), molecular dynamics (MD), surface plasmon resonance (SPR), virtual screening (VS).

*De novo* discovery of conformer-stabilizing VHHs relies on the mining of *in vivo* matured or synthetic repertoires with high diversity, using powerful *in vitro* enrichment methodologies, mainly phage or yeast surface display (YSD) (Figure 2).

#### Panning of *In Vivo* Matured and Synthetic VHH Repertoires to Discover Type I and Type II ConfoBodies

To successfully identify Type I Cbs that mimic G proteins from *in vivo* matured repertoires, purified agonist-bound receptors reconstituted into liposomes were required as the immunogen (Figure 2). Near-milligram quantities of the native receptor were used to perform a Cb discovery cycle, enabling immunization and repertoire selections. In order to mine these *in vivo* matured repertoires, iterative rounds of selection on an agonist-bound purified receptor in liposomes or detergent micelles were performed (Figure 2). Depletions with antagonist-occupied receptors were regularly performed. For effective depletion against inactive state binders, one of the receptor conformers should be occupied with ligands with receptor residency times approaching those of covalent ligands (Kruse et al., 2013). The advantage of reconstituting agonist-occupied receptors into liposomes, particularly for immunizations, is threefold: 1) it is expected to favor the presentation of a large population of agonist-bound native receptors by skewing the equilibrium toward ligand-bound receptors; 2) it improves the receptor’s stability upon injection into the camelid; 3) it allows the presentation of the intracellular loops of the agonist-occupied receptor to the animal’s immune system. Following camelid immunizations, active state-stabilizing Type I Cbs Nb80/Nb71, Nb39, Nb9-8, and Nb7 have been reported against ADRB2, OPRM, ACM2, and US28, respectively (Supplementary Table S1). As a consequence of the high amino acid sequence conservation of the Nb80 epitope between human ADRB2 and ADRB1, Nb80 is not only cross-reactive but also stabilizes the active state of human ADRB1. Similarly, OPRM Nb39 stabilizes the active state of its paralogue OPRK. The affinity of the agonists used in these successful Cb discovery campaigns (BI167107 for ADRB2; Dmt1-Dalda for OPRM; iperoxo or covalent derivative FAUC123 for ACM2; CX3CL1 for US28) was low-nM to near covalent, as assessed on the basal receptor conformation (Rasmussen et al., 2011a; Kruse et al., 2013; Burg et al., 2015; Huang et al., 2015). To identify Type II Cbs Nb60 (ADRB2) and Nb6 (OPRK), *in vivo* matured VHH repertoires obtained after immunization with agonist-occupied liposomes were mined. Whether agonist or antagonist/inverse agonist-occupied receptor samples were used for panning was not described (Staus et al., 2014; Che et al., 2018). The strategy to identify Nanobody6 (SUCR1) was not described in detail (Haffke et al., 2019).

Active state stabilizing, G protein-mimicking Type I Cbs against ADRB2, AGTR1, SMO, and AA2AR were also identified by screening a synthetic VHH repertoire (Supplementary Table S1). Mining synthetic VHH repertoires avoids laborious and time-consuming immunizations. Furthermore, synthetic libraries are not compromised by immune tolerance, immunization-driven epitope bias, or seroconversion to denatured receptors caused by antigen instability (Zimmermann et al., 2018). A synthetic VHH repertoire was designed by randomizing the CDRs of a consensus framework scaffold based on llama germline genes (McMahon et al., 2018). Yeast surface display, combining iterative rounds of magnetic-activated cell sorting (MACS) and minimally one round of fluorescence-activated cell sorting (FACS), enabled the identification of Type I active state-stabilizing Nb.c202 against ADRB2, Nb.AT110 against AGTR1, NbSmo8 against SMO, and Nb.AD101/102 against AA2AR. Counter selection to remove inactive state GPCR binders by applying inverse agonist-occupied receptor was reported to be critical. Additionally, receptor preparations labeled with different fluorophores in consecutive selection rounds were used to remove accidental binders to the fluorophores. Compared to phage display, one of the major advantages of YSD is the ability to deep-mine (i.e., identify ultra-low frequency events) the VHH repertoire by single event sorting of VHH displaying yeast cells with particular characteristics. Indeed, by differential fluorescent staining of the agonist- and antagonist-occupied receptor, YSD allows positive sorting of those yeast cells that preferentially interact with the agonist-bound receptor.

#### Affinity Maturation to Engineer High-Affinity ConfoBody Variants

AGTR1 Type I Cb Nb.AT110, mined from the synthetic repertoire described above, was subjected to *in vitro* maturation resulting in higher affinity Cb variant Nb.AT110i1 (Wingler et al., 2019). Nb.AT110i1 was used to obtain the active state AGTR1 protein structure bound to low (near µM) affinity agonists TRV055 (Wingler et al., 2019). Compared to the parent Cb NbAT110, the *in vitro* matured Cb NbAT110i1 induced a more pronounced increase in affinity of agonist TRV055 for the Cb-stabilized AGTR1 (pK_i__parental Cb_ = −6.58 *vs.* pK_i matured Cb_ = −7.56) and further improved TRV055 affinity for the active *versus* the basal state of the receptor: 245-fold for the affinity matured Cb (Table 2) versus 26-fold for the parent Cb Nb.AT110 (Wingler et al., 2019). In a separate publication, Nb.AT110 was also successfully matured *via* directed evolution by autonomous hypermutation in yeast cells (Wellner et al., 2021; Table 2). Compared to the parent Cb, the matured Cb Nb.AT110i103 showed a 20-fold improved affinity to the agonist-bound receptor. Whether the latter *in vitro* evolution approach enables the *de novo* identification of the active state GPCR Cbs approach remains to be demonstrated. ADRB2 Type I Cb Nb80 was affinity matured to obtain Cb 6B9 (Table 2; Ring et al., 2013). Cb 6B9 was used to obtain the adrenaline-occupied ADRB2 active state structure (Ring et al., 2013).

**TABLE 2:** Examples and characteristics of affinity matured Type I ConfoBodies. For uniformity in GPCR nomenclature in this review, the GPCR synonym of the UniProt database is the one indicated in Figure 4. Abbreviations: yeast surface display (YSD), detergent soluble (DS).

GPCR	Parent Cb	Affinity matured Cb (introduced # mutations)	Affinity maturation method	Agonist affinity improvement (Cb_parental_ *vs.* Cb_matured_)^a^	Agonist Affinity Improvement (Cb_matured_ active state *vs*. basal state)^a^	Reference
ADRB2	Nb80	6B9 (#8: S30A, I31L, T33I, S56T, Y100F, V103I, L104I, E106D)	Randomized Nb80 by error prone PCR	10x (BI167107)—assesses by single cycle kinetics SPR on DS ADRB2 –	Adrenaline: 504	Ring et al. (2013)
			YSD and BI167107-bound DS ADRB2			
AGTR1	Nb.AT110	Nb.AT110i1 (#4: A31V, N58D, I98V, Y113N)	YSD, error prone PCR	1x (AngII)	AngII: 6	Wingler et al. (2019)
			AngII occupied DS AGTR1	10x (TRV055)	TRV055: 245	
AGTR1	Nb.AT110	Nb.AT110i103^b^ (#3: R66H, I98V, Y113H)	YSD (directed evolution by autonomous hypermutation using AngII occupied DS AGTR1)	Not indicated	Not indicated^c^	Wellner et al. (2021)

a Determined by the gold standard radioligand competition assay (see text), unless otherwise mentioned.

b Additional affinity matured active state-stabilizing VHHs (Nb.AT110i101-103) are reported in Wellner et al. (2021).

c To demonstrate the active state stabilization of the affinity matured Cb, the authors demonstrated a 20-fold improved binding (reduced IC_50_) of Nb.AT110i103 in the presence and absence of agonist TRV055 in a radioligand competition assay displacing antagonist radioligand [3H]-olmesartan with a serial dilution of the Cb (Wellner et al., 2021).

#### Panning of *In Vivo* Matured VHH Repertoires to Identify Transducer-Stabilizing ConfoBodies

Three Type III Cbs which stabilize transducers have been reported (Table 1; Supplementary Table S1). Gs protein stabilizing Cb (Nb35) was identified from an *in vivo* matured VHH repertoire following immunization with a BI167107 occupied, cross-linked ADRB2:Gs ternary complex (Rasmussen et al., 2011b). Two rounds of biopanning against a cross-linked ADRB2:Gs:BI167107 ternary complex (solid-phase immobilized or in solution with complex, reconstituted into biotinylated high-density lipoprotein particles) resulted in the discovery of Nb35. Its active state-stabilizing behavior is demonstrated *via* size exclusion chromatography, revealing that Nb35 protects the ADRB2:Gs:BI167107 complex from dissociation by GTPγS. As a crystallographic chaperone, Nb35 helped to obtain the G protein-bound active state ADRB2 protein structure by interacting with an interface of the Gαs and Gβ subunits of the heterotrimeric Gs protein (Rasmussen et al., 2011b), stabilizing the nucleotide-free Gs.

A different approach was taken by English and co-authors (2019), who developed an elegant *in vitro* directed evolution display method in mammalian cells to mine for cytosolically expressed VHHs that engage the 5HT2A receptor’s signaling state by phenotypic screening. The authors co-expressed a GPCR, a signaling sensitive fluorescence reporter system and an *in vivo* matured VHH repertoire directed towards purified 5HT2A in a mammalian cell background. By transducing a stable cell line expressing the fluorescence reporter gene and the GPCR with recombinant virus enabling expression of the VHH repertoire (with a multiplicity of infection <1), the authors aimed for a single VHH expression per mammalian cell. In order to identify Cb VGS-Nb2, fluorescent cells were sorted with high reporter gene activation. Although VGS-Nb2 improved the affinity of the small molecule agonist DOI (Knight et al., 2004) to 5HT2A, a substantial population of the receptor remained in the low-affinity state (Figure 5E in English et al., 2019). Whether this was due to the affinity of the VHH, its mode of interaction with the receptor:Gq complex, or assay conditions is not clear. Despite indirect evidence that VGS-Nb2 does not bind to the interface of a 5HT2A conformer coupled to a signal transducer (English et al., 2019), further characterization will be required to confirm how VGS-Nb2 interacts with serotonin receptor 5HT2A and whether VGS-Nb2 is a fully active state transducer-stabilizing Type III Cb similar to Nb35.

Nb32, which stabilizes β-arrestin, was identified using a cross-linked GPCR:β-arrestin complex. The complex consists of a chimeric ADRB2-V2R receptor, containing the ADRB2 and the C-terminal tail of vasopressin 2 receptor (V2R), β-arrestin-1, and a β-arrestin-1-specific Fab (Fab30). The cross-linked complex was used for immunization and subsequent phage display, alternating solid phase immobilization in the first round and biopanning in solution in the second round. Nb32 binds to a β-arrestin epitope that is only accessible when β-arrestin is in complex with the GPCR, as Nb32 does not bind the individual components of the complex. Nb32 stabilizes the GPCR:β-arrestin complex, resulting in an increased population of the β-arrestin “core” conformation, as visualized by negative-stain electron microscopy. This “core” conformation is a marker of G protein desensitization and is assumed to represent the active state GPCR:β-arrestin complex (Cahill et al., 2017; Nguyen et al., 2019).

### Assays to Confirm Conformer Specificity of VHHs

Multiple assays can be deployed to demonstrate whether GPCR-specific VHHs are conformer-stabilizing. These assays rely on the fact that conformer-stabilizing VHHs help stabilize the cytosolic pocket of the GPCR in an active (Type I and Type III Cbs) or inactive (Type II Cbs) conformation. The VHHs behave as positive or negative allosteric modulators, i.e., they bind to an epitope distinct from the orthosteric binding pocket and modulate the affinity of an agonist ligand for its GPCR target. These assays can be grouped into the following assay classes based on four distinct assay principles: 1) the modulation of the ligand affinity to the Cb-occupied GPCR; 2) the modulation of the Cb binding to the ligand-occupied GPCR; 3) the ligand selective recruitment of the Cb to the GPCR-biosensor; and 4) the GPCR transmembrane α-helical movement (summarized in Supplementary Table S2).

**FIGURE 3:**
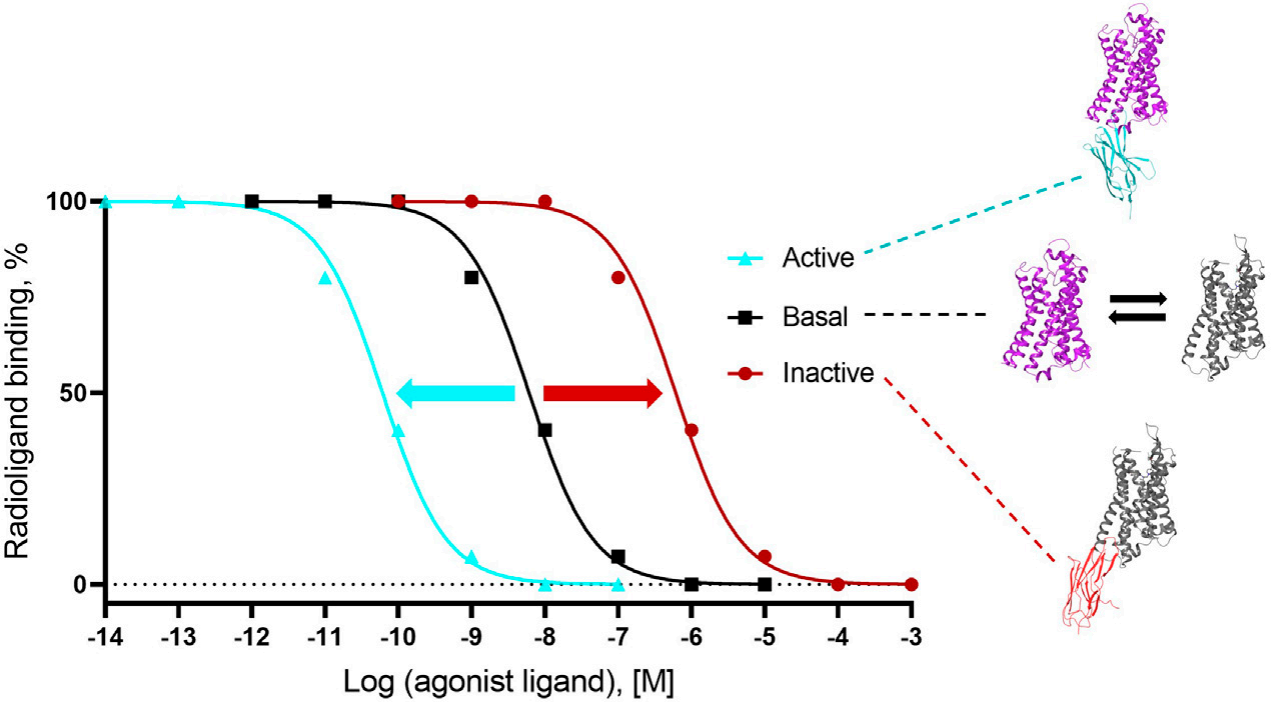
Gold standard assay to confirm conformer sensitivity of VHHs (illustration of the potential impact of a ConfoBody). The assay uses a neutral antagonist radioligand that is agnostic for the distinct receptor conformers (i.e., shows an identical affinity for all conformers) and a cold agonist competitor. Following dose-dependent competition with a cold agonist in the presence of excess amounts of the conformer-stabilizing VHH, IC_50_ values of the cold agonist can be calculated. A ConfoBody that stabilizes the active state receptor conformer will increase, similar to the G protein (not shown), the affinity of the competing cold agonist ligand for the GPCR (i.e., reduces the IC_50_ of the agonist for the receptor) compared to the affinity of the agonist for the basal conformer (absence of Cb or presence of irrelevant VHH). In contrast, a Cb that stabilizes the inactive state receptor conformer will decrease the affinity of the competing agonist ligand for the GPCR (i.e., increases the IC_50_ of the agonist for the GPCR) compared to the affinity of the agonist for the basal conformer. A Cb that causes a significant leftward or rightward shift of the curve compared to the curve obtained with the basal receptor conformation is either an active state- or inactive state-stabilizing, Type I or II Cb, respectively.

For Type I and Type II G protein-mimicking Cbs, the “Gold standard” assay to demonstrate conformer stabilization is the radioligand competition assay, which measures the modulation of the inhibition constant (K_i_ as a measure of affinity) of a cold agonist competitor to the radio-labeled antagonist ligand (Figure 3) on a Cb-occupied receptor. Such assays have been established with cells (or derivatives, including membranes) that overexpress the GPCR of interest or with a purified receptor (detergent soluble or reconstituted into liposomes or nanodiscs; see references in Supplementary Table S2). If the affinity of the cold agonist to VHH-doped GPCR is significantly modulated *versus* the condition without VHH, then the VHH is either an active state or inactive state-stabilizing, Type I or II Cb (Figure 3). Examples of the “Gold standard” assay for Type I, Type II, and Type III Cbs are published by Rasmussen et al. (2011a); English et al. (2019), and Che et al. (2020), respectively.

Alternative assays to the radioligand competition assay have been deployed by multiple authors to assess the selectivity of a Cb for a particular GPCR conformer or to further characterize Cbs. One panel of assays quantifies the agonist-ligand affinity to Cb-occupied receptor via agonist radioligand binding (Staus et al., 2014). The second group of assays detects differential binding of the Cb to apo or ligand-occupied receptor: ELISA, flow cytometry, pull down, size exclusion chromatography, and surface plasmon resonance (Staus et al., 2014; McMahon et al., 2018; Wingler et al., 2019; Deshpande et al., 2019). The use of ligands with different pharmacology (agonist, antagonist, and inverse agonist) that favor different GPCR conformers is crucial to confirm the conformer-stabilizing behavior of VHHs.

Biosensor assays are *in cellulo* assays that monitor the translocation of the cytosolically expressed Cb to the receptor (or the receptor:transducer complex) upon ligand incubation. Fluorescent (Irannejad et al., 2013) and bioluminescence resonance energy transfer-based read-outs have been deployed (Che et al., 2020).

The transmembrane movement assay, is based on fluorescence emission spectra using monobromobimane labeled GPCR and monitors the intramolecular change in distance between critical transmembrane α-helices (Rasmussen et al., 2011a).

References to the assays above-described that have been used to show conformation selectivity for Type III Cbs can be found in Supplementary Table S2.

## Structurally Enabling GPCRs With ConfoBodies

**FIGURE 4:**
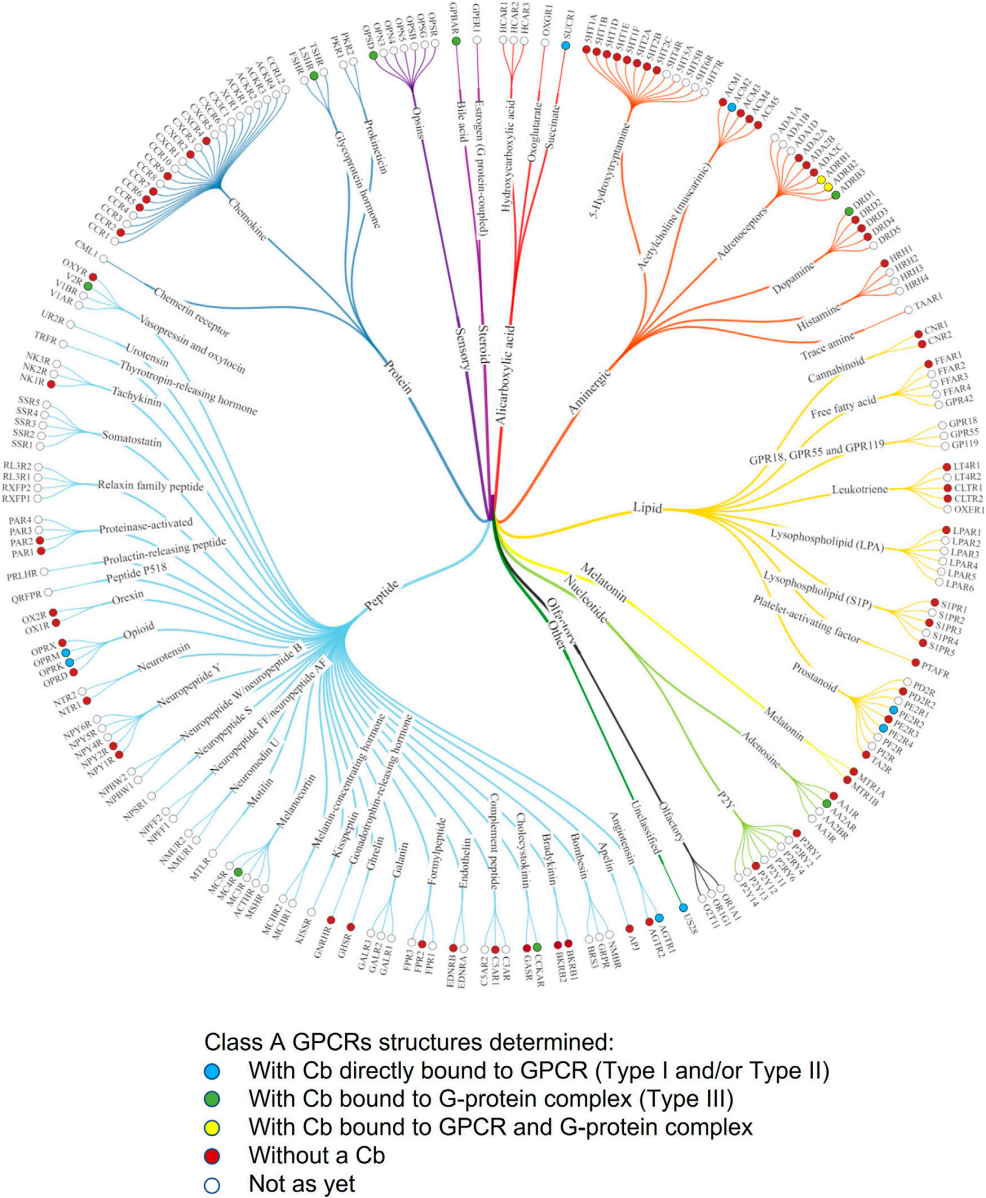
Phylogenetic tree of Class A GPCRs indicating GPCR structures that have been solved with ConfoBodies. Receptors are referred to by their UniProt gene name. Figure adapted from the phylogenetic structural coverage plot for Class A GPCRs from GPCRdb, which excludes the orphan GPR52 receptor that was solved using a Type III Cb (Pándy-Szekeres et al., 2021). Structures captured up until 13.12.2021.

This section presents an overview of the GPCR structures that were solved using ConfoBodies (Types I, II, and III Cbs, as defined in Table 1) and discusses how these tools have revolutionized the structural understanding of GPCRs. Out of 188 GPCR structures deposited in the PDB to date, 118 were solved with the aid of protein chaperones (biologics and derivatives thereof). Of these, 114 were determined with Type I, II, or III Cbs (Figure 4 and Supplementary Tables S3, S4) in the presence of an agonist, inverse agonist, antagonist, an agonist and allosteric modulator together, as well as in their apo form. The binding of a chaperone to a target protein typically increases the target protein’s polar surface area, which may lead to an increase in favorable protein-protein interactions between protein-chaperone entities within the solution. Ultimately this may help facilitate crystal lattice formation, a phenomenon critical for successful X-ray crystallography studies (Hino et al., 2013). These chaperones also increase the effective size and asymmetry of the studied GPCR, which, in turn, can make the receptor more amenable to Cryo-EM studies. It must be noted that the use of Cbs does not tackle the major hurdle in the GPCR structural biology field, namely, the purification of native GPCRs in sufficient quantities for structure determination.

Interestingly, of these 114 Cb-enabled GPCR structures, 109 have the hallmarks of active state structures, which is remarkable given the elusive nature of GPCR active state conformations. In fact, bovine rhodopsin was the first GPCR to be structurally determined in active and inactive conformational states (Park et al., 2008; Scheerer et al., 2008). In rhodopsin, the light-induced transition from the inactive to the active state is very efficient. Agonists, however, are much less efficient at stabilizing the active state of GPCRs (Rasmussen et al., 2011a). The first agonist-bound fully active human GPCR structure was solved by X-ray crystallography using a Type I Cb (Rasmussen et al., 2011a), and this was followed shortly thereafter by the first X-ray structure of a G protein:GPCR complex, solved *via* the use of a Type III Cb (Rasmussen et al., 2011b). This work heralded the start of a new and exciting era for GPCR structural biology. Indeed, prior to the availability of these unique tools, the Kobilka laboratory had attempted to use derivatives of conventional antibodies, such as Fab and single-chain variable (scFv) fragments, as chaperones to aid membrane protein structure determination. However, these were of limited use due to the generation of inactive-like structures representing the basal states (Rasmussen et al., 2007).

**FIGURE 5:**
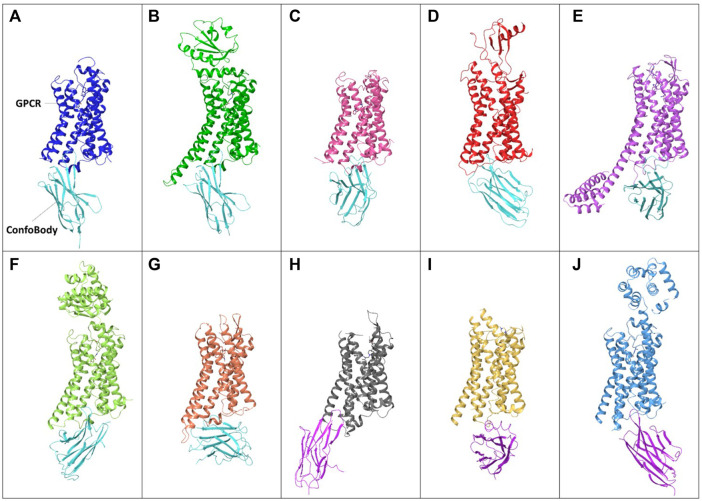
Examples of unique published ConfoBodies used to solve Class A GPCR active state structures (depicted by cyan ConfoBodies) and inactive state structures (depicted by magenta ConfoBodies). **(A)** Nb80 with ADRB2 (blue) PDB = 3P0G. **(B)** Nb6B9 with ADRB1 (green) PDB = 6H7N. **(C)** Nb9-8 with ACM2 (pink) PDB = 4MQS. **(D)** Nb7 with US28 (red) PDB = 4XT1. **(E)** Nb.AT110i1 with AGTR1 (magenta) PDB = 6OS2. **(F)** Nb71 with ADRB2 (lime) PDB = 6MXT. **(G)** Nb39 with OPRM1 (orange) PDB = 5C1M. **(H)** Nb6* with OPRK1 (silver) PDB = 6VI4. **(I)** Nanobody6* with SUCR1 (yellow) PDB = 6RNK. **(J)** Nb60 with ADRB2 (light blue) PDB = 5JQH. *There are two Nb6 ConfoBodies deposited which are different. All GPCRs are structurally aligned to the backbone of the 3P0G GPCR structure.

Camelid VHHs have three hypervariable loops corresponding to the complementarity determining loop regions (CDRs) 1, 2, and 3, which contribute almost exclusively to antigen recognition. These CDRs can adopt many shapes, such as convex or concave paratopes, unlike conventional antibody fragments (Desmyter et al., 1996). Such paratopes enable VHHs to recognize protein surface clefts and cavities and confer the ability to bind non-linear 3D epitopes, such as those presented by the different conformational states induced by agonists and antagonists/inverse agonists. These qualities enable VHHs to bind and successfully stabilize diverse GPCRs in distinct conformational states, as exemplified by the numerous active and inactive state structures (Figure 5 and Supplementary Tables S3, S4) that have been solved with the aid of Cbs and will be discussed further in the following section. Similar to receptor engineered constructs that have been used to solve GPCR structures, it cannot be excluded that the use of Cb chaperones may introduce some GPCR structural artifacts.

### Type I and Type II Cbs, Unique Tools Facilitating GPCR Structure Determination

Besides enabling drug screening or pathway deconvolution (described in section below) one of the most prominent applications of ConfoBodies is their use as chaperones in GPCR structural biology studies. Cbs were the first “tools” to enable agonist-bound active state GPCR structure determination. Early work carried out by the Kobilka lab in collaboration with the Steyaert lab, delivered the ADRB2 structure in complex with a high-affinity ligand and Nb80 (Rasmussen et al., 2011a). This first fully active state agonist-bound GPCR structure revealed the extent of conformational changes associated with Class A activation: comparison with the inactive state ADRB2 structure revealed that subtle changes in the ligand binding pocket triggered an 11Å outward movement of the cytoplasmic end of TM6, along with rearrangements of TM5 and TM7. Rhodopsin was found to undergo similar structural changes upon light activation which helped to verify that the agonist-bound ADRB2 Nb80 structure had adopted an active conformational state. Through emerging structures, it became apparent that these conformational changes are hallmarks of GPCR activation, and that ConfoBodies can perfectly mimic G protein binding, by recognizing and stabilizing active state conformers. Following this initial landmark structure, multiple GPCR active state structures were solved using X-ray crystallography with the aid of diverse Type I Cbs: AGTR1, ADRB1, ACM2, OPRM, US28 (Class A GPCRs), and SMO (Class F GPCR) (Figure 5 Supplementary Tables S3, S4).

ConfoBody-enabled GPCR structures not only helped to delineate orthosteric ligand binding sites presented by GPCRs in their active state but also gave much needed insight into positive allosteric modulator (PAM) binding pockets. For instance, Kruse demonstrated that the allosteric binding site is revealed and largely pre-formed in the presence of an agonist and Cb. This was achieved by comparing the Type I Cb Nb9-8 complexed active state structures of ACM2 solved in the presence of the agonist iperoxo on the one hand and the positive allosteric modulator LY2119620 on the other hand (Kruse et al., 2013). In addition, unique insights into partial agonism or biased activation were provided by the Type I ConfoBody Nb71 in complex with ADRB2 and the long-acting partial agonist Salmeterol. Structural comparison with ADRB2 bound to the full agonist epinephrine revealed differences in the hydrogen-bond network involving residues Ser204 and Asn293 (Masureel et al., 2018), which explained the difference in the recruitment of G protein and β-arrestin between the partial and full agonists.

The determination of active state structures with endogenous ligands by affinity matured Cbs has further demonstrated the power of Cbs as structural biology chaperones. Cb 6B9, an affinity matured derivative of Nb80 (Table 2), was instrumental in acquiring an active state structure of ADRB2 in complex with the endogenous low-affinity small molecule agonist adrenaline. Additionally, the chaperone Cb NbAT110i1, which is an affinity matured derivative of NbAT110 (Table 2), enabled the determination of the active state AGTR1 structure in complex with the endogenous partial agonist peptide AngII.

Type II Cbs has also been successfully used to determine inactive state class A GPCR structures such as ADRB2 with Nb60 (Staus et al., 2016), SUCR1 complexed with Nanobody6 (Haffke et al., 2019), and OPRK using Nb6 (Che et al., 2020) (Figure 5 and Supplementary Tables S3, S4).

### Class A GPCR Structural Insights Afforded Using Type III Cbs

The successful applications of Type I Cbs described above were key for understanding GPCR signaling at a structural level and for gaining insights into the binding of different types of activators. However, the use of the Type III Cb Nb35 truly revolutionized our knowledge of the G protein-bound active state conformations adopted by GPCRs (see Supplementary Tables S3, S4).

The first X-ray crystal structure of a GPCR:G protein:Nb35 complex was reported in 2011 for ADRB2 (Figure 1; Rasmussen et al., 2011b). Nb35 was found to bind at the interface between the Ras-domain of Gαs and Gβ, with CDR1 interacting mainly with Gβ, and CDR3 interacting with both Gβ and Gαs. Nb35 prevents GTPγS mediated dissociation of the nucleotide-free complex (Livingston et al., 2018; Maeda et al., 2018). Although Nb35 was first used to solve an X-ray crystal structure of ADRB2, it has since proven invaluable as a tool for enabling the study of other active state GPCRs, by helping to stabilize scores of GPCR:G protein complexes (Figure 4 and Supplementary Tables S3, S4).

The insights brought by the numerous Class A GPCR structures have been crucial for advancing our understanding of GPCR pharmacology and enabling GPCR drug discovery. Firstly, Nb35 helped reveal and delineate agonist binding pockets of many class A GPCRs (Figure 4 and Supplementary Tables S3, S4), paramount for structure-based drug design. Secondly, G protein complexed active state structures have also deepened our understanding of receptor biology. For instance, the active state structure of V2R complexed with Gs protein helped to explain how the V2R R137H or R137 L/C variants could lead to two severe genetic diseases (Bous et al., 2021), and the MC4R structure helped to explain how Ca^2+^ is required for agonist, but not antagonist efficacy (Israeli et al., 2021).

Arguably one of the most interesting class A GPCR structures solved with Nb35 is the GPCR:G protein:β-arrestin megacomplex. This structure was solved using two Cbs with Nb35 bound to the G protein heterotrimer and Nb32 recognizing a specific conformation of β-arrestin in the presence of Fab30 (Nguyen et al., 2019). The Lefkowitz group’s structure revealed the remarkable simultaneous engagement of G protein with the GPCR and of β-arrestin to the phosphorylated C-terminus of an active state human chimeric ADRB2 receptor-bearing the C-terminal tail of V2R (β2V2R) (Nguyen et al., 2019). These findings ultimately provided a structural basis for sustained G protein signaling after internalization of the GPCR, a phenomenon reported for some GPCRs such as V2R and PTHR (Ferrandon et al., 2009; Feinstein et al., 2013; Pavlos and Friedman, 2017).

### Type III Cb Use Beyond Class A GPCRs

To date, 81 out of the 162 structures of fully activated GPCR:G protein complexes that were deposited in the PDB have been solved with the aid of Type III Cb Nb35, with the majority solved via Cryo-EM (Supplementary Tables S3, S4). These structures include important class A drug targets, as well as those of class B and class F GPCRs, demonstrating the importance of Nb35 for advancing our appreciation of GPCR structural biology across the classes.

Class B GPCR structures, solved in complex with Gs protein with the aid of Nb35, have revealed that the active state conformations differ from those observed for Class A GPCRs. The structural hallmark of class B GPCR activation is the more pronounced outward shift of TM6, relative to class A GPCRs, and the concomitant formation of a sharp kink in the middle of TM6, which is induced and stabilized by agonist binding (Liang et al., 2017). Structural studies enabled by Nb35 also elucidated the mechanism of activation of class B GPCRs by endogenous peptide ligands and the role of the ectodomains (ECD). For instance, the functional analysis demonstrated that the PAC1R ECD behaves as an affinity trap and is not required for receptor activation (Kabayashi et al., 2020). Conversely, the GLP1R ECD plays an indispensable role in receptor activation, highlighting the functional diversity of different ECDs in class B GPCRs (Zhang et al., 2017). Class B structures also revealed additional allosteric modulator binding site insights. Bueno et al. determined the structure of the GLP1R bound to LSN3160440 in complex with GLP-1 and heterotrimeric Gs (Bueno et al., 2020). This allosteric modulator was found to bind high in the helical bundle at an interface between TM1 and TM2, allowing access to the peptide ligand.

A fascinating application of Cbs in structural biology was recently described (Qiao et al., 2020). In this study, the basis of G protein specificity was elucidated, and new insights into the molecular details that govern pleiotropic GPCR:G protein coupling were obtained for a class B GPCR. The authors determined structures of the human glucagon receptor (GCGR) bound to glucagon and the distinct heterotrimeric G proteins, Gs and Gi. Different chaperones were used to enable these structural elucidation efforts: Nb35 for Gs and scFv16 for Gi (as scFv16 can stabilize Gi: in addition to Gq:GPCR complexes) (Qiao et al., 2020). These two structures present a similar open binding pocket to accommodate the Gs and Gi proteins. The less bulky Gi protein is accommodated in the large intracellular cavity but forms less extensive, predominantly hydrophobic interactions, accounting for G protein coupling specificity. In contrast, the Gs binding selectivity of GCGR is explained by a larger interaction interface.

### Extending the Utility of the Type III Cb Nb35

Other strategies exploiting Nb35 as a protein structure chaperone have been pursued. For instance, a more stable complex was generated to facilitate the purification of the G protein using an engineered G protein, containing a mini-G_S_ protein, a βγ subunit, and Nb35 instead of the full G protein heterotrimer (García-Nafría et al., 2018). The Nb35 epitope is retained in the mini-G_S_:βγ heterotrimer. Mini-G proteins are engineered GTPase domains of Gα subunits, which bind to GPCRs and recapitulate the increased agonist affinity observed upon coupling of a native heterotrimeric G protein (Nehmé et al., 2017).

Engineered dominant-negative (DN) Gαs subunits have been used along with Nb35 to enhance the formation of active GPCR:G protein complexes for Cryo-EM structural studies, towards receptors where this has not been possible using wild-type Gs protein (Liang et al., 2018a). An engineered DN Gαs subunit has a reduced nucleotide affinity which ultimately limits Gα:Gβγ dissociation and consequently enhances the stability of the agonist:GPCR:G protein heterotrimeric complex. Using DN Gαs and Cb35 tools, Liang et al., 2018b determined the active state structure of the elusive class B GPCR CALRL complexed with the sensory neuropeptide agonist CGRP and the essential accessory protein RAMP1.

Another strategy applied to the determination of recent structures involves using chimeric G protein, whereupon the G protein retains both Nb35 and scFv16 chaperone epitopes of the G protein heterotrimer. This strategy helped to obtain the first structure of GLP1R with a small molecule partial agonist, LY3502970, which is biased toward G protein over β-arrestin (Kawai et al., 2020). The high-resolution structure revealed a unique binding pocket in the upper helical bundle where the compound was found to bind to the ECD *via* extracellular loop 2 and TM helices 1, 2, 3, and 7. The binding of the compound created a distinct receptor conformation that may explain the partial agonism and biased signaling invoked by this ligand.

Furthermore, the combination of Nb35 with NanoBit technology has been recently described (Duan et al., 2020). Inspired by the complementation principle of NanoBiT^2^ technology (Promega), the authors fused the SmBiT peptide at the C-terminus of the Gβ subunit to bind the LgBiT that was attached to the C-terminus of the truncated receptor. This provided an extra linkage to stabilize the interface of the receptor and the G protein in addition to Nb35. This structure provided insights into the molecular basis of PACAP27 (the ligand of VIP1R) binding and VIP receptor activation, thereby enabling structure-based drug discovery approaches.

### Molecular Modeling of Cb-Stabilized Conformations Advances the Understanding of GPCR Structure and Dynamics

In order to support GPCR structure elucidation efforts *via* crystallography, atomistic molecular dynamics (MD) simulations can be performed to increase the confidence of the proposed crystallographic structure. This was exemplified by the recent Cb-enabled active state crystal structure of DRD1, solved with an agonist, for which there was limited ligand resolution (Sun et al., 2021). Some GPCRs have high intrinsic flexibilities that preclude the use of crystallogenesis approaches. Cryo-EM can elucidate structures of such challenging membrane proteins, as evidenced by the growing list of GPCR:G protein complexed cryo-EM structures that have been determined (summarized in Supplementary Table S3).

However, some GPCR structures are particularly challenging to determine in their calculated cryo-EM maps. Computational and biophysical approaches were developed in conjunction with the use of conformationally stabilizing ConfoBodies to overcome this. One such approach employs a novel hybrid strategy that was developed by Bous et al. (2021), where MD simulations were combined with saturation transfer difference (STD) nuclear magnetic resonance (NMR) to help build unambiguous structural models of ternary complexes in the cryo-EM maps. Using the combination of this approach and Nb35, two distinct states of the wild-type AVP hormone-bound V2R—with heterotrimeric Gs‐protein—were determined. This provided unprecedented molecular insights into the mechanism of G protein activation by V2R. The authors commented that the conformational heterogeneity observed in these cryo-EM studies was made possible by using a native receptor.

The study of the conformational states of GPCRs is useful in increasing the understanding of the structural changes that culminate in GPCR activation or for the elucidation of ligand-competent states. However, distinct conformational states are not always observable *via* biophysical experiments (Martí-Solano et al., 2016). MD simulations can provide insights into the structural ensembles of GPCRs and the underlying dynamics. To observe such phenomena that occur over micro to millisecond timescales, one needs access to dedicated supercomputers or special-purpose hardware. In one study, using an MD-dedicated machine, it was possible with classical MD to study the inactivation mechanism of ADRB2, starting from the Nb80 stabilized active state structure. However, it was not possible to capture activation (Dror et al., 2011).

Enhanced sampling methods have been used in conjunction with MD to further study the conformational landscapes of GPCRs. In one such study performed by Kohlhoff et al. (2014), MD simulations that were initiated from inactive and active ADRB2 structures were performed using a volunteer-distributed computing platform Folding@home. The resultant MD trajectories were then “stitched” together with Markov state model (MSM) to capture rare events. In another study performed by Lovera et al. (2019), MSM was applied to analyze MD simulations of an *apo* AA2AR inactive state structure generated using high throughput MD and adaptive sampling. In both studies, through the use of and/or comparison to Cb-stabilized active state structures, it became apparent that activation and deactivation may proceed through multiple pathways, visiting several distinct metastable intermediate states. Kohlhoff et al.’s study also showed that ligands act by modulating the receptor dynamics to favor different pathways and ultimately different populated states. Lovera et al.’s study further revealed that the active state of AA2AR is minimally populated in the conformational energy landscape, which is consistent with the concept of GPCR basal activity. Furthermore, compared with the available Gs-Nb35 active state structures of AA2AR, it was proposed that conformational selection and induced fit could play a role in G protein binding, which in turn may help explain why a single G protein can bind many GPCRs. In summary, molecular modeling studies of Cb-enabled structures have helped charter the conformational landscapes of GPCRs.

### ConfoBody-Enabled Structure-Based Drug Design

The availability of three-dimensional structures of GPCRs in their inactive states has led to the extensive use of structure-based drug design (SBDD) to discover small molecules, namely, antagonists and inverse agonists, that bind to this conformation (Kooistra et al., 2013; Andrews et al., 2014). In contrast, the use of SBDD to discover ligands that bind to GPCR active states has been limited. The recent increase in active state GPCR structures obtained through the use of ConfoBodies (Figure 1) and the conformational “snapshots” derived from those using MD have significantly enabled GPCR agonist discovery and development efforts.

Recent studies have shown the importance of these active state structures for enabling the discovery of agonists using *in silico* techniques such as docking and virtual screening (VS). Costanzi and Vilar (2012) retrospectively looked at the impact of using ADRB2 structures solved in different conformations for their ability to discriminate agonists from antagonists and decoys. The authors used four different ADRB2 structures, each solved with a ligand: one active state (3P0G, with a full agonist and Nb80 bound) and three inactive state structures (2RH1, with an inverse agonist-bound; 3NYA, with a neutral antagonist-bound; and 3PDS, with an irreversible agonist-bound). In total, 30 agonists, 30 antagonists, and ∼60,000 decoys were docked, and the ability of each structure to discriminate between the ligands was measured. It was observed that the inactive state structures with an inverse agonist or antagonist-bound (2RH1 and 3NYA) were able to discriminate antagonists from agonists. The active state structure (3P0G) displayed an even stronger bias toward agonists over antagonists. Interestingly, the last structure, with an inactive-like state and an irreversible agonist-bound (3PDS), could not discriminate between agonists and antagonists. All structures were, however, able to properly discriminate binders from the 60,000 decoys used in this study. A comparable exercise carried out by Scharf et al. (2019) led to similar findings. In addition, it was concluded that the use of multiple active state structures is beneficial for the retrieval of agonists.

Other studies also used structures solved with the aid of Cbs. Redji et al. (2019) used a newly described GLP1R active state structure to discover a novel positive allosteric modulator (PAM). The same structure was used by Latek et al. (2019) in a drug-repurposing effort. They identified several commercial drugs *via* docking into the active state of GLP1R that show promise as type 2 diabetes drugs. Miao et al. (2016) used an active state structure of the ACM2 receptor to perform a series of MD simulations to generate an ensemble of active state-derived binding pockets, against which they performed a small VS study. From the 38 selected compounds, three were confirmed to be PAMs of the ACM2 receptor. In another study, Weiss et al. (2013) used the ADRB2 structure, solved with Nb80 (PDB: 3P0G), to perform a prospective VS campaign, resulting in the discovery of two novel and distinct scaffolds that demonstrated agonistic activation of the receptor. The potential for using this ADRB2 structure as a template for generating an active state homology model of DRD2 was then explored. Unfortunately, VS against the resultant model only yielded two weak (µM) agonists and produced overall hit rates and potencies similar to those acquired using an inactive DRD3 structure as a template. This highlights the importance of solving the structures of receptors in their active state to effectively enable SBDD against a desired GPCR target.

The increasing availability of receptor structures in multiple conformations and the advance in fields such as proteo-chemometrics and artificial intelligence allow the development of methodologies that can go beyond those outlined above. While current SBDD approaches focus on predicting ligand affinities, new methods are emerging that aim to predict the function of ligands. Kooistra et al. (2015) exemplified this using protein-ligand interaction fingerprints (IFP) in order to predict the modality of various ADRB1/2 ligands.

## ConfoBody-Enabled Drug Discovery and Pathway Deconvolution

Many drug targets adopt different functional conformations, thereby increasing the challenges and hurdles for drug discovery. GPCRs have lower affinities for agonists in the basal states compared to their G protein-bound active state conformations. Moreover, different agonists can stabilize distinct receptor conformations. Consequently, not all cellular signaling pathways linked to a receptor (pharmacological fingerprint) are uniformly activated by any agonist. As outlined above, Cbs can distinguish between receptors bound to (partial) agonists, antagonists, and inverse agonists (Figure 2), in artificial overexpressing systems and primary cells (Stoeber et al., 2018). Thus, Cbs are employed as versatile tools to study signal transduction and understand the structure-function relationships of the receptor conformers. This will be illustrated in the examples below.

### ConfoBodies to Study Signaling Pathway Dynamics

Cbs are excellent biosensors; the unique single-domain nature of a VHH allows for functional expression in the reducing cytoplasmic environment as a fusion with a fluorescent protein moiety, allowing for sub-cellular tracking. Enhanced GFP-fused Nb80 was used to probe the ADRB2 active state conformer in living mammalian cells (Irannejad et al., 2013). ADRB2 activation by the agonist isoprenaline was observed not only at the plasma membrane but also on the early endosome membrane. Interestingly, this activation came in two waves. Upon addition of the agonist, eGFP-Nb80 was rapidly recruited to the plasma membrane, and ADRB2 was subsequently internalized within minutes. The second phase of Nb80 recruitment started acting on the internalized receptors. These findings were confirmed by monitoring Nb37 recruitment and cAMP signaling data (Irannejad et al., 2013). Nb37, a Type III Cb, is a specific biosensor of Gs activation as it binds selectively to the GDP-free Gαs subunit (Supplementary Table S1). Nb37 can thus be used to detect the cellular location of the GPCR:Gs signaling complex. For instance, Sungkaworn et al. (2017) used eYFP-labelled Nb37 to visualize the activation of ADRB2 in live cells, demonstrating that GPCR activation preferentially happens in hot spots on the cell membrane. Similarly, eGFP-labelled Nb33 (a Type I Cb to OPRM, OPRD, and OPRK, Supplementary Table S1) has been used to reveal a location bias of OPRM and OPRD agonists (Stoeber et al., 2018). By using eGPF-Nb33, it was demonstrated that small molecule agonists and endogenous peptides differ in the subcellular location at which they activate OPRM. While cell-impermeable peptides activate at the plasma membrane and propagate to endosomes after ligand-induced internalization, cell-permeable small molecules such as morphine drive an extra wave of OPRM activation in the Golgi apparatus. This endosomal signaling was demonstrated to be ligand-dependent by reversion with a cell-permeable antagonist (naloxone) but not by a cell-impermeable antagonist or ligand washout. This was confirmed in overexpressing HEK293 cells and primary rat striatal neurons. In neurons, even endogenous OPRM could be detected using eGFP-Nb33. Contrary to the work on ADRB2, no impact of Nb33 on OPRM mediated cAMP accumulation was observed for OPRM under the conditions tested.

Conformation-stabilizing VHHs also allow dissection of the GPCR signaling pathways by modulating the interaction of the receptor with signal transducers. A panel of Type I and Type II ADRB2 Cbs was evaluated for the impact on receptor signaling (Staus et al., 2014). These Cbs can differentially block the downstream effectors of the activated receptor (G protein activation, GRK-mediated receptor phosphorylation, and β-arrestin recruitment). Inactivation of receptor signaling seemed to be either *via* stabilization of an inactive conformation by Type II Cbs or *via* steric blocking of the effector by some of the Type I Cbs. The relative differences in G protein or β-arrestin effector blockade by Type I Cbs suggest that some of these ConfoBodies (e.g., Nb63 and Nb72) inhibit β-arrestin more than G protein recruitment, while others (e.g., Nb65, Nb80, and Nb82) similarly inhibit G protein and β-arrestin recruitment (Staus et al., 2014). The difficulty of interpreting the differential modulation of the Cbs on downstream effector functions could be attributed to effector blockade by Cb-enabled conformer stabilization or sterically blocking transducer complexation.

### ConfoBodies to Dissect Ligand Pharmacology

Cbs have also been used to dissect ligand pharmacology, providing evidence that GPCRs do not act through a single active and inactive state but rather through multiple active and inactive receptor states that are differentially stabilized by various ligands. Staus et al. (2014, 2016) profiled a panel of ADRB2 agonists, inverse agonist, and antagonists by analyzing their binding to ADRB2 in the presence of either Type I Cb Nb80 (positive allosteric modulator) or Type II Cb Nb60 (negative allosteric modulator; Supplementary Table S1). Cooperativity values (α) were determined for both Cb Types I and II. While these were clear-cut for the full agonists (*α* > 1 for Nb80 and α < 1 for Nb60), the relationship turned out to be more complex for the partial agonists. Interestingly, the inverse agonist-bound ADRB2-preferring Nb60 reduced the affinity of the high-affinity agonist isoprenaline by approximately 70-fold compared to the “inactive” (VHH-free) state, indicating that the VHH-free receptor experiences an average of various inactive states (DeVree et al., 2016).

A similar exercise was carried out using OPRK and Type I Cb Nb39 on the one hand and Type II Cb Nb6 on the other hand. Labeled Nb39 and Nb6 were used as biosensors in live cells to investigate ligand behavior. Using a BRET assay, clear dissociation of Nb6 and association of Nb39 was demonstrated upon agonist application (Che et al., 2020). This concept was further extended to class A GPCRs coupling to the different G protein families by making chimeric receptors with grafted OPRK ICL3, the binding site of Nb6 (El Daibani and Che, 2021). In a similar fashion, Vasudevan et al. confirmed agonist-selective recruitment of cytosolically expressed Nb39 to OPRM by means of the NanoBiT^®^ technology (Vasudevan and Stove, 2020). Upon activation of the receptor with a set of synthetic opioids (fentanyl, fentanyl analogues, and a non-fentanyl opioid U-47700), the active state sensor Nb39 was recruited, which enabled the pharmacology of the ligands to be studied while monitoring their respective OPRM activation effects (Vasudevan and Stove, 2020). Livingston et al. described the use of an interferometry-based technique to determine the intrinsic efficacy of orthosteric agonists and positive allosteric modulators of the OPRM using the active state sensor Nb39 and purified monomeric OPRM reconstituted in high-density lipoprotein particles (rHDL). rHDL particles were immobilized on an interferometry probe, after which the probe was exposed to saturating concentrations of ligands and a sub-saturating concentration of Nb39 (1 µM). In the absence of ligand, no detectable binding of Nb39 was observed, indicating low levels of the constitutively active receptor. Conversely, pre-incubation of the rHDL particles with a wide range of agonists (e.g., methadone, loperamide, and PZM21), both peptides and small molecules, led to the binding of Nb39–albeit to varying degrees. Orthosteric antagonists such as naloxone or diprenorphine failed to promote detectable Nb39 binding to OPRM. Like orthosteric agonists, allosteric ligands BMS-986187 and BMS-986122 also stimulated Nb39 binding to the OPRM, even in the absence of an orthosteric ligand but to a lesser extent. This technique has proven to be more sensitive than traditional measures of efficacy (e.g., GTPγ^35^S binding assay) and does not rely on signal amplification (Livingston et al., 2018).

### ConfoBody-Enabled Small Molecule Screening

**FIGURE 6:**
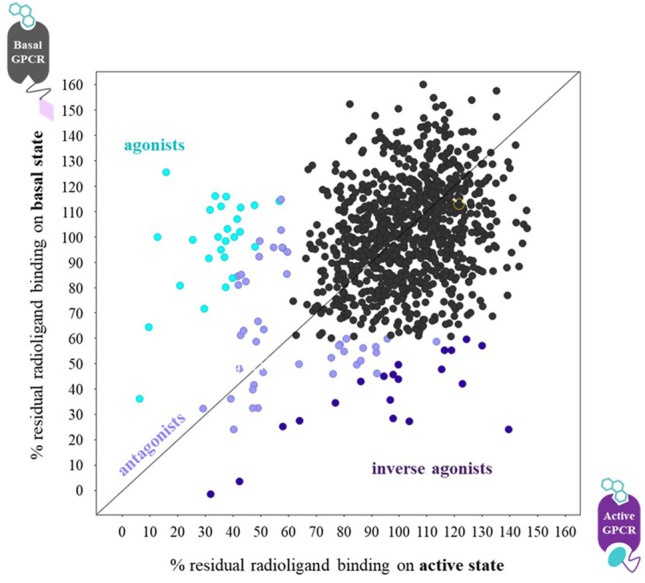
Graph of the radioligand assay principle when performing parallel fragment screens on a ConfoBody-fused GPCR (● = Cb80), representing its active state, and the same receptor fused to an irrelevant nanobody (♦ = irr VHH), representing its basal state conformation. Each data point measures the residual binding of the radiolabeled antagonist to the receptor. Agonists, antagonists, and inverse agonists differentially displace the radiolabeled antagonist. The ConfoBody-fused GPCR stabilizes a high-affinity agonist state, hence increasing the affinity for agonist-like fragments leading to a higher radioligand displacement on the active state conformation. Neutral antagonist fragments bind the basal and active state equally well (thus displace radioligand equally well independent of receptor conformer), while inverse agonists preferentially bind the basal state conformation, hence showing a higher radioligand displacement on the basal state conformation. Figure adapted from Pardon et al. (2018).

As demonstrated by the examples above, Cbs serve as attractive tools to study, modulate, and exploit highly dynamic and challenging GPCRs (Mujić-Delić et al., 2014; Heukers et al., 2019; Uchański et al., 2020). Aside from the ability to allow for structural determination and pharmacological modulation of receptor activity (agonism, antagonism, and inverse agonism), G protein-mimicking Type I Cbs enable the discovery of novel small molecules that selectively bind the Cb-stabilized GPCR conformer. Pardon et al. (2018) described comparative fragment screens against a ADRB2-Nb80 fusion (representing active state ADRB2) and the same receptor fused to an irrelevant VHH (Nbirr) (representing basal-state ADRB2). The binding preference of reference ligands (agonist epinephrine, neutral antagonist alprenolol, and inverse agonist ICI 118,551) to one GPCR conformer over the other demonstrated that measuring changes in ligand-binding affinity for distinct conformational states can be used to predict the pharmacology of a compound in a system-independent manner. The fragment-based screen was performed using ADRB2-Nb80 and ADRB2-Nbirr fusions in a single-point radioligand displacement assay. This led to the identification and ranking of novel orthosteric fragments that preferentially bind to the active state of the receptor (ADRB2-Nb80 fusion). These fragments thus exhibited a similar conformer preference as known ADRB2 agonists (assay principle shown in Figure 6). Fragments exhibiting the highest selectivity and considerable ligand efficacy for the ADRB2-Nb80 fusion were further elaborated in a structure-based manner, supported by molecular docking and computational tools. Consecutive optimization rounds eventually yielded sub-nanomolar affinity compounds for ADRB2-Nb80, showing functional activity in a cellular ADRB2 mediated cAMP assay.

## Discussion and Future Perspectives

As outlined in the previous sections, Cbs have been used as tools to support screening campaigns for active state ligands and facilitate GPCR active state structure elucidation efforts, which have enabled computer-aided drug discovery efforts (Figure 2). Cbs achieve this by exquisitely stabilizing distinct conformers of GPCRs during signal transduction. Cbs recognize and bind distinct conformers presented by wildtype receptors isolated from the membrane (in micelle, nanodisc, and related formats). More importantly, Cbs can stabilize physiological receptor conformations *in cellulo*, thus enabling the study of ligand or compound pharmacology under more physiologically relevant conditions compared to those when the GPCR is extracted from its natural lipid environment.

In this section, we will discuss future perspectives of Cb-enabled drug discovery, focusing on 1) the exploration of novel strategies for *de novo* Cb discovery and the application of conformation-stabilizing Cbs targeting extracellular GPCR epitopes, 2) the engineering of GPCRs to extend the application of existing Cbs toward other GPCRs; 3) the engineering of GPCRs and Cbs to facilitate cryo-EM GPCR structures; 4) the potential of Cb-enabled SBDD and screening to support the discovery and development of conformer-selective small molecules and biologics with desired pharmacology; and 5) whether the stabilization of desired conformers may help unleash the therapeutic potential of other protein classes, by targeting disease-relevant conformational states.

### Exploring Novel Strategies for *De Novo* Cb Discovery and Targeting Extracellular GPCR Epitopes With Conformation-Stabilizing Cbs

So far, to the best of our knowledge, the only antibody-derived scaffolds reported to fully stabilize active state conformations by cooperatively and directly interacting with the intracellular pocket of GPCRs are VHHs (G protein-mimicking Type I Cbs). Indeed only inactive state-stabilizing antibodies against intracellular epitopes of GPCRs are reported for conventional antibodies and fragments thereof (Hino et al., 2012). The only example of a synthetic, non-antibody scaffold is an aptamer that can mimic a G protein (Kahsai et al., 2016).

Since the identification of the first active state Cbs in 2011, Type I and Type II Cbs have been generated against six Class A GPCR receptor families (prostanoid, adenosine, adrenoceptor, angiotensin, opioid, acetylcholine, and succinate), as well as the unclassified Class A GPCR US28 (Supplementary Table S1) and the Class F smoothened (SMO) receptor (Figure 4 and Supplementary Table S1). Besides the Gs-stabilizing Nb35, two additional Type III Cbs have been reported: VGS-Nb2 toward Gq and Nb32 toward β-arrestin. Despite the tremendous progress in GPCR biochemistry over the last 10 years (exemplified by the rapidly increasing number of active state GPCR structures in the PDB), purifying native GPCRs remains labor-intensive. Therefore, the *de novo* discovery of conformer-stabilizing reagents for GPCRs (including antibodies or alternative scaffolds), which consumes significant quantities of the isolated receptor, remains a challenge. Strategies that could make Cb discovery more efficient, for example, by reducing the need for or by becoming fully independent of purified protein, will be discussed in addition to the potential use of conformation-stabilizing Cbs to extracellular epitopes of GPCRs in drug discovery.

For *de novo* Cb discovery, multiple discovery strategies were reported to successfully identify active state Cbs (Figure 1 and Supplementary Table S1), applying distinct recombinant display methodologies (yeast, ribosome, and mammalian display) to mine *in vivo* matured and synthetic repertoires. Still, without exception, all strategies rely on access to the native purified receptor. Therefore, purified protein-free strategies to identify active state Cbs would represent a major breakthrough. The use of proteomes that harbor GPCRs, such as detergent-solubilized lysates, overexpressing cells, or cell derivatives for immunization or selection, remains to be further explored. Additionally, Cb discovery strategies that dramatically reduce the consumption of purified protein are appealing. *De novo* Cb discovery strategies *via* YSD of a synthetic VHH repertoire avoided laborious camelid immunizations. This resulted in the identification of Cbs toward AGTR1, ADRB2, AA2AR, and SMO (Wingler et al., 2019; McMahon et al., 2018; Deshpande et al., 2019). The diversity of this synthetic library (∼1E8) is rather low (McMahon et al., 2018). Consequently, one may expect the need for affinity maturation of the resulting Cbs for downstream applications where high-affinity VHHs are desired (e.g., structural biology). A positive correlation exists between the library diversity and improved affinity (lower K_D_) of *de novo* identified antibodies of this repertoire (Ponsel et al., 2011). Indeed, out of the Cbs identified from this synthetic repertoire (Nb.AT110, Nb.c200, and NbSmo8; Supplementary Table S1), Cb-enabled agonist-bound active state structures were reported for SMO (no affinity maturation required; Deshpande et al., 2019) and AGTR1 (following affinity maturation of parental Cb Nb.AT110; Wingler et al., 2019). Another synthetic VHH repertoire with diversity ∼1E12 was designed by Zimmermann et al. (2018). Although the authors did not describe the delivery of GPCR conformer-stabilizing VHHs, the mining of such ultra-large synthetic VHH repertoires did result in the identification of conformer-selective VHHs for other classes of integral membrane proteins with single-digit nM affinities (Zimmermann et al., 2018). According to the authors, to fully exploit the repertoire diversity of these ultra-large synthetic VHH libraries (diversities beyond 1E10 are nearly impossible to display on yeast because of low transformation efficiencies), the combination of a single round of ribosome display and subsequent rounds of phage display was critical. Following this strategy, an extracellular OX2R binder was identified (Sb51), bound to the agonist-occupied OX2R with sub-nM affinity (Hong et al., 2021). Sb51 was used as a chaperone to determine an active state OX2R structure in the presence of a small molecule agonist. While not reported in this article, the authors disclosed during a conference (Discovery on Target, Boston, September 2021) that Sb51 improved the affinity of the small molecule agonist against the receptor 10-fold. Sb51 thus stabilizes the OX2R active state conformer through a positive allosteric-modulating mechanism *via* extracellular epitopes of the receptor (EC PAM). Similar examples of EC PAMs against class C mGluR2 and mGluR5 that exhibit improved binding for agonist-occupied receptors have also been reported (DN10 and DN13 to mGluR2 in Scholler et al. (2017); Nb43 to mGluR5 in Koehl et al. (2019)). It remains to be determined whether such EC PAMs can be used to screen for orthosteric small molecules with agonist pharmacology (beyond their use as chaperones to generate active state structures) or whether such PAMs can be developed as therapeutic candidates in their own right.

As the custom identification of GPCR-specific Cbs remains time-consuming and labor-intensive, the discovery of additional Type III Cbs that can stabilize the active state of multiple receptors merits further exploration. Further characterization of VGS-Nb2 (Supplementary Table S1; English et al., 2019), a potential GPCR:Gq complex-stabilizing Cb will help ascertain whether VGS-Nb2 can be used to determine the structures of Gq-coupled receptors, in an analogous fashion to Gs-coupled receptors stabilized by Nb35. If so, the discovery strategy published by English et al. (2019) could be extended to identify Cbs that stabilize additional GPCR:transducer complexes. An approach that enables the mining of Cbs from synthetic VHH repertoires *via* similar phenotypic screens represents an attractive prospect for finding Cbs without the need for purified protein.

An effort to engineer the “off-the-shelf” ADRB2 Type I Cb, Nb80, toward the wild type CCR7 receptor by randomizing the Cb’s antigen binding loops was reported (Jakobs et al., 2019). The target GPCR (CCR7), fused to one domain of split YFP, was co-expressed with the randomized Nb80 repertoire fused to the complementary part of YFP in mammalian cells. Following incubation with the agonist and subsequent sorting of agonist-induced fluorescent cells (YFP complementation), the authors attempted to identify Type I Cbs against CCR7. Although no Type I Cbs were identified against CCR7, the authors did discover VHHs that could bind CCR7 (albeit they were cross-reactive with ADRB2).

### Engineering GPCRs to Extend the Application of Existing Cbs Toward Other GPCRs

As an alternative to *de novo* Cb discovery, different methods to extend “off-the-shelf” Cbs of Types I, II, and III to stabilize conformers of other GPCRs have been sought. The concept of stabilizing GPCR conformers by designing chimeric receptors that recognize and bind existing Cbs is an attractive approach. This strategy has been successfully described in studies that graft the intracellular loop 3 (ICL3) of the OPRK (the epitope for Type II inactive state-stabilizing Cb Nb6) to acceptor GPCRs (Che et al., 2020). For two GPCR chimeras (5HT2A and ET_A_), the addition of an agonist selectively dissociated Nb6 from the chimera, a strong indicator that the inactive state-stabilizing behavior of Nb6 can be extended beyond OPRK. A similar strategy extended the stabilization propensity of Type I Cbs toward active state chimeric GPCRs (De Blieck et al., 2020). The use of Cbs to stabilize GPCR chimeras in particular conformations has been described in the patent WO2020221768 (De Blieck et al., 2020). It remains to be further investigated whether a generic approach of such engineered chimeric receptors will represent the native folding of the stabilized conformation and whether signal transduction will be impacted.

### Engineering GPCRs and Cbs to Facilitate Cryo-EM GPCR Structures

Despite recent progress in cryo-EM, resulting in high-resolution protein structures of GPCRs in complex with Type III transducer-stabilizing Cbs, no peer-reviewed work is available that describes Type I or Type II Cb-enabled cryo-EM structures. However, it was recently reported in an unreviewed article that the use of Type II Cbs is sufficient for solving GPCR structures *via* cryo-EM (Robertson et al., 2021)^3^. In order to help solve additional inactive state structures, the GPCR-intracellular epitope of Nb6 was interchanged from OPRK to other GPCRs by grafting in the OPRK intracellular loop 3 to GPCRs of interest. Nb6 retained its ability to bind with high affinity to the resultant chimeric GPCRs (Tichy et al., 2019), resulting in the generation of the inactive state structures of SST2 and NTR1 at a high resolution *via* cryo-EM, whose structures have yet to be made available in the PDB.

A possible option to exploit Type I Cbs in cryo-EM structure determination studies may be to increase the molecular weight of the chaperone in the GPCR:Cb complex. MegaBodies™ (Mb) are VHHs that have been engineered to increase the size and asymmetry of VHHs (Laverty et al., 2019; Uchański et al., 2020). Using this approach, a VHH is grafted onto a large protein scaffold to increase the VHH’s molecular weight, while enabling the VHH to retain its affinity and selectivity for the intended target. Two scaffold proteins have been used thus far: the adhesin domain of *H. pylori* (HopQ) and the Glucosidase YgjK of *E. coli* K12. The MegaBody approach has been successfully used for other membrane proteins in cryo-EM but not yet for Cb-stabilized active state GPCRs.

### New Avenues to Advance the Potential of Cb-Enabled Drug Discovery for Small Molecules and Antibodies

Given the recent progress in GPCR structural biology, to a large extent enabled by Cbs, GPCR structures are anticipated to become increasingly available in the earlier stages of the drug discovery campaigns (hit to lead phase).

Consequently, structure-informed rational design of small molecules will gain traction as a key approach for rapidly and efficiently developing hits into quality leads. Recent studies have revealed the importance of such active state structures for enabling the discovery of agonists using *in silico* techniques such as docking and VS (described above). By using such *in silico* approaches, GPCR structures can also be used to anticipate “off-target” side effects (Latek et al., 2019), considerations that become increasingly important during lead development.

The principle of using conformer-stabilizing VHHs becomes of particular interest in view of the emerging concept that one can selectively modulate GPCR-induced signaling pathways by means of biased ligands (Smith et al., 2018). By binding to distinct GPCR conformations, biased ligands may selectively modulate downstream signaling pathways, as such minimizing activation of pathways that cause adverse side effects in some diseases. Although high-resolution structures of different receptor states are available, atomistic details of allosteric signaling across the membrane remain elusive. Initiating atomistic MD simulations under physiologically relevant conditions using GPCR coordinates derived from the active state ADRB2 structure (solved in complex with the Type I Cb Nb80), Fleetwood et al. (2020) revealed that it is possible to characterize locally connected “microswitches” that communicate agonist binding with the intracellular region. Furthermore, they used this structural information to develop artificial intelligence models (neural networks) that reveal the complex network of communication in play between the different receptor states. Studying the influence of ligands on the microswitches and predicting the influence on the equilibrium between conformational states represents a key step in designing ligands with biased signaling properties and paves the way toward more effective drugs. The identification of Cbs that selectively improve the agonist affinity of biased ligands could help further elucidate the difference in structure-activity relationship between agonist- and biased agonist-bound active state structures to further examine particular signaling pathways and their roles in different disease states.

One Cb-enabled fragment screening example that delivered conformer selective molecules against GPCRs is described in the peer-reviewed literature (Pardon et al., 2018), based on radioligand displacement. The high sensitivity of such a Cb-stabilized screening assay is ideal for the identification of chemical starting points from fragment libraries, giving access to substantially more chemical diversity compared to classical HTS compound libraries. Despite the high potential of such a screening assay to identify new chemical entities for challenging GPCRs, a radioligand is not always available. Alternatively, non-radiometric Cb-enabled screening assays based on proximity assay techniques have been successfully developed (Menet et al., 2020) and were presented during the 2021 7½th RSC/SCI symposium on GPCRs in Medicinal Chemistry conference^4^ (United Kingdom., 24–25 February, 2021, “The Confo Therapeutics Technology Platform Enabling GPCR Fragment-based Drug”). These conformation-selective screening assays were highly sensitive, enabling the successful screening of fragment libraries. However, they are also suited for high throughput screening.

In addition to Cb-enabled SBDD and conformer-selective screening for small molecules, some publications advocate that conformational drug discovery can be extended toward structure-guided rational design and *de novo* identification of conformer-selective antibodies (Ma et al., 2020; Hong et al., 2021). Using (conformer-stabilizing) VHHs toward extracellular epitopes of GPCRs as structural chaperones can be of similar interest as Cbs of Types I, II, and III. Few structures exist of active state VHH:GPCR receptor complexes using VHH chaperones that interact with extracellular epitopes of the GPCR. The Sb51:cmpd1:OX2R active state structure is one such example (Hong et al., 2021). Despite not being described by Hong et al. (2021), Sb51 was explicitly presented by the authors as a PAM during the 2021 Discovery on Target conference (Boston, 27 to 30 September 2021, “Structures of Active State Orexin Receptor 2 Rationalize Peptide and Small-Molecule Agonist Recognition and Receptor Activation”). The structure could be used to guide the elaboration of small molecule cmpd1 into more potent or selective agonist leads. Another example of a VHH that interacts with the extracellular epitope of a GPCR is JN241 against the APJ receptor (Ma et al., 2020). The antagonist JN241:APJ co-structure was critical for the rational structure-guided design of an agonistic VHH derivative JN241-9, achieved by engineering very subtle residue changes in the binding cavity (Ma et al., 2020). Molecular modeling and simulation of wild-type APJ with antagonist JN241 and agonist JN241-9 revealed different binding modes. Notably, JN241-9 was found to mimic the peptide agonist AMG3054 binding to APJ, suggesting that the strategy for converting antagonists into agonistic VHHs may be more readily applied to GPCRs, for which peptidic agonists are known. This engineering strategy resembles an elaboration effort of a small molecule or peptide modification where relatively small changes can influence and change the ligand’s pharmacology. Reports on *de novo* discovery of agonistic antibody fragments to GPCRs are rare: DN10 for mGluR2 (Scholler et al., 2017) and JN300 for APJ (Ren et al., 2020), confirming the challenge of discovering GPCR agonist biologics. The unique potential of Cbs to facilitate *de novo* discovery of agonist VHHs to human GPCRs was presented during the 2021 Discovery on Target conference (Boston, 27 to 30 September 2021, “Stabilizing GPCRs in Their Therapeutically Relevant Conformation to Discover Therapeutic Antibodies”). Cb-stabilized active GPCR conformers were presented as crucial reagents (Laeremans, 2021) to identify agonistic VHHs with nM *in vitro* potencies from *in vivo* matured VHH repertoires by phage display.

### Conformational Drug Discovery Beyond GPCRs

The principle of using conformation-stabilizing VHHs in drug discovery could be applied to all drug target classes that undergo conformational changes (Lawson, 2012). Protein structures using conformer-stabilizing VHHs against other druggable integral membrane proteins such as growth factor receptors (Nevoltris et al., 2015), ion channels (Zimmermann et al., 2018; Sigoillot et al., 2019), and transporters (Zimmermann et al., 2018) have been obtained, revealing previously occluded allosteric druggable pockets.

## Conclusion and Perspectives

Tremendous progress has been made in recent years to understand the structure-activity relationship between ligand pharmacology and GPCR conformations during signal transduction, often leveraged by the use of Cbs. Cb-enabled active state structures have revealed elusive conformer-specific structural features of ortho- and allosteric extracellular druggable pockets. Cbs have since been used to establish sensitive screening assays for conformer-selective fragment and small molecule hit-finding campaigns, resulting in molecules with desired *in vitro* pharmacology and potency. The exciting next hurdle is to find out whether such newly developed conformer-selective chemical or biological entities can be developed into transformative therapeutics with superior clinical efficacy in human subjects. Many GPCRs remain therapeutically untapped due to a lack of understanding of their role in disease and the lack of target-specific modulators. The use of ConfoBodies may help further our understanding of GPCR biology by dissecting the signaling pathways that link disease conformations to target related pathologies. Expanding the toolbox for conformational drug discovery while further integrating structure-guided and computer-aided drug design with conformer-specific drug screening will confirm whether conformer-stabilizing VHH technology can be expanded toward other drug target classes where conformational changes play a role in target induced signaling pathways linked to pathologies.

## Footnotes

1 “ConfoBody” and “ConfoBodies” are registered trademarks of Confo Therapeutics NV.

2
https://www.promega.com/resources/technologies/nanoluc-luciferase-enzyme/?cs=y.

3
https://www.biorxiv.org/content/10.1101/2021.11.02.466983v1.full.

4
https://www.rsc.org/events/detail/41236/7-th-rsc-sci-symposium-on-gpcrs-in-medicinal-chemistry.
