# Supplementary material for: Accelerating GPCR Drug Discovery With Conformation-Stabilizing VHHs
Source: Front Mol Biosci. 2022 May 23;9:863099. doi: 10.3389/fmolb.2022.863099 (PMC9170359; doi:10.3389/fmolb.2022.863099)
Supplement: Supplementary file 1 [file DataSheet1.docx]

**SUPPLEMENTARY TABLES**

**Supplementary Table S1.** Discovery specifics of conformer-stabilizing VHHs (ConfoBodies). For uniformity in GPCR nomenclature in this review, the GPCR synonym of the Uniprot database chosen is the one indicated in Fig. 4.

| **Conformer -stabilizing VHH** | **Target** | **ConfoBody type^**^** | **Repertoire source (immunogen)** | **Display method (GPCR format)** | **Fold modulation of agonist affinity^*^** | **References** |  |
| --- | --- | --- | --- | --- | --- | --- | --- |
| Nb80^a^ | ADRB2 | I | *In vivo* matured (BI167107 doped LS) | Phage display (BI167107 doped LS) | Isoproterenol: 95 | Rasmussen et al., (2011a)  Ring et al., (2013)  McMahon et al., (2018) |  |
| Nb80 and  6B9 | ADRB1 | I^b^ | -^c^ | -^c^ | Not reported for ADRB1 | Warne et al., (2019)  Liu et al., (2019) |  |
| Nb71^a^ | ADRB2 | I | *In vivo* matured (BI167107 doped LS) | Phage display (BI167107 doped LS) | Salmeterol: 2.6  Isoproterenol: 1.8 | Masureel et al., (2018) |  |
| Nb60^d^ | ADRB2 | II | *In vivo* matured (BI167107 doped LS) | Phage display (GPCR format not described) | Fenoterol: 0.25^e^  Isoproterenol: 0.014 | Staus et al., (2014)  Staus et al., (2016)  DeVree et al., 2016 |  |
| Nb.c200^f^ | ADRB2 | I | Synthetic (NA) | YSD (BI167107-bound DS receptor) | Adrenaline: >100^e^ | McMahon et al., (2018) |  |
| Nb9-8 | ACM2 | I | *In vivo* matured (Iperoxo doped LS) | YSD (FAUC123-bound DS receptor) | Iperoxo: >100^e^ | Kruse et al., (2013) |  |
| Nb39^g^ | OPRM | I | *In vivo* matured (Dmt1-Dalda doped LS) | Phage display (Dmt1-DALDA doped LS) | BU72: 29  DAMGO: 84  Endomorphin-2: 24 | Huang et al., (2015) |  |
| Nb39 | OPRK | I | -^h^ | -^h^ | Dynorphin A (1-17): 6 | Che et al., (2020) |  |
| Nb6^i^ | OPRK | II | *In vivo* matured (SalA doped LS) | Phage display (LS, not specified whether agonist occupied receptor was used) | Dynorphin A (1-17): 0.1 | Che et al., (2018)  Che et al., (2020) |  |
| Nb7 | US28 | I^j^ | *In vivo* matured (CX3CL1 doped LS) | YSD (CX3CL1 occupied DS receptor) | Not reported | Burg et al., (2015) |  |
| VUN103 | US28 | I^j^ | See Nb7 | Phage display (membrane extracts of HEK293 or U251 over expressing US28) | Not reported | De Groof et al., (2021) |  |
| Nb.AT110 | AGTR1 | I | Synthetic (NA) | YSD (AngII-doped DS receptor) | AngII: 6 | Wingler et al., (2019) |  |
| Nb.AD101  Nb.AD102 | AA2AR | I^k^ | Synthetic (NA) | YSD (UK 432097-doped DS receptor) | Not reported | McMahon et al., (2018) |  |
| NbSmo8 | SMO | I^l^ | Synthetic (NA) | YSD (SAG21k-occupied DS receptor) | Not reported | Deshpande et al., (2019) |  |
| Nanobody6 | SUCR1 | II^m^ | Not described | Not described | Not reported | Haffke et al., (2019) |  |
| Nb35 | Gs (in complex with agonist occupied GPCR) | III^n^ | In vivo matured (cross-linked complex) | Phage display (BI167107 occupied cross-linked DS receptor complex or BI167107 occupied nanodisc reconstituted receptor complex) | Not reported | Rasmussen et al., (2011b) |  |
| Nb37 | Idem as Nb35 | III^n^ | Idem as Nb35 | Idem as Nb35 | Not reported | Westfield et al., (2017)  Rasmussen et al., (2011b)  Irranejad et al., (2013) |  |
| VGS-Nb2 | Gq (in complex 5HT2A) | III | *In vivo* matured (LSD doped LS) | Mammalian display (ligand independent directed evolution by autonomous hypermutation in mammalian host overexpressing 5-HT_2A_) | DOI: > 100^e, o^ | English et al., (2019) | |
| Nb32 | β-arrestin (in complex with agonist and GPCR) | III^p^ | In vivo matured (cross-linked complex) | Phage display (BI167107 occupied cross-linked DS receptor / β-arrestin / Fab30 complex) | Not reported | Cahill et al., (2017)  Nguyen et al., (2019) | |

Abbreviations: GPCR reconstituted in liposomes (LS); Yeast surface display (YSD); detergent soluble (DS)

^*^Compared to the affinity for the basal receptor conformer as determined by the gold standard radioligand competition assay (Fig.5), unless otherwise mentioned.

^**^The categorization of Cbs is described in Table 1.

^a^Other but less characterized active state stabilizing VHHs (all Type I Cbs) are categorized based on CDR3 AA sequence clustering in Staus et al., (2014): Nb64 and Nb65 are classified as representatives of the Nb80 Cluster B; Nb82, Nb83, Nb84 and Nb72 belong to Cluster C; Nb63, Nb67, Nb69, Nb71 and Nb86 belong to a panel of divergent clusters.

^b^Based on protein structure of Nb80 or 6B9 in complex with agonist occupied ADRB1.

^c^Due to Nb80 epitope conservation between ADRB1 and ADRB2, Nb80 also stabilizes active state conformer of ADRB1.

^d^NbA11, a conformer-stabilizing VHH that recognizes inactive state ADRB2 (Type II), similar to Nb60, is reported in Staus et al., 2016.

^e^Number estimated from graph.

^f^Additional Type I active state stabilizing VHHs reported in McMahon et al., (2018) belonging to divergent clusters (Nb.c201-203).

^g^An additional Type I active state stabilizing VHHs Nb33 is reported in Sounier et al., (2015) belonging to the same sequence cluster as Nb39.

^h^Due to Nb39 epitope conservation between OPRM, OPRD and OPRK, Nb33 (a Type I Cb belonging to the same sequence cluster as Nb39) also stabilizes active state conformer of OPRK and OPRD (Stoeber et al., 2018).

^i^Due to Nb6 epitope conservation between OPRM, OPRD, OPRX (nociception receptor) and OPRK, Nb6 also stabilizes inactive state conformers of OPRD, OPRX and OPRK (Che et al., 2020).

^j^Classification as Type I based on protein structure. While both Nb7 and VUN-103 interact with intracellular epitopes of the constitutively activated US28 receptor, VUN-103 interacts with both apo and CX3CL1 occupied receptor, contrary to Nb7 which preferably binds to CX3CL1 occupied receptor. Consequently, Nb7 and VUN-100 seem to interact with different conformers with subtle differences.

^k^Selectivity for agonist occupied receptor demonstrated via flow cytometry (comparative yeast cell staining with agonist and antagonist occupied receptor) and pull-down assay.

^l^Type I classification based on protein structure.

^m^Based on [^35^S]GTPγS assay data and Nanobody6 complexed antagonist occupied SUCR1 protein structure.

^n^Active state conformer stabilization is demonstrated by Cb enabled inhibition of GTP induced receptor-Gs complex dissociation.

^o^Stabilizing ~50% of the 5HT2A population in the high affinity Gαq-coupled state.

^p^Increasing the percentage of GPCR*β-arrestin complexes in ‘core’ conformation from 34 to 63% based on negative-stain electron microscopy.

**6Supplementary Table S2.** Assays deployed to demonstrate conformer-stabilizing behavior of VHHs. For uniformity in GPCR nomenclature in this review, the GPCR synonym of the Uniprot database chosen is the one indicated in Fig. 4.

| **Assay principle** | **Assay description** | **ConfoBody example and reference** |
| --- | --- | --- |
| I. Modulation of ligand affinity to GPCR by Cb | *Radioligand - competition.* This assay is considered as the gold standard assay in case antagonist radioligand is available. It measures the affinity (IC_50_ and derived inhibitory constant Ki) of cold ligand for (VHH occupied) receptor. Can be applied with purified receptor or receptor in natural (overexpressing cells or derived membrane extracts) or artificial (nanodisc) membrane matrix. A VHH is conformer-stabilizing when the affinity of the cold agonist to the GPCR in excess of the candidate conformer-stabilizing VHH is significantly modulated versus the condition without a VHH (or a dummy VHH). A VHH that causes a decreased (leftward shifted) or increased (rightward shifted) IC_50_ is an active state or inactive state stabilizing VHH, respectively (see Figure 3). | **Type I**  Nb80 (ADRB2; Rasmussen et al.,2011a)  Nb6B9 (ADRB2; Ring et al., 2013)  Nb80/Nb71 (ADRB2; Masureel et al., 2018)  Nb.c200 (ADRB2; McMahon et al., 2018)  Nb9-8 (ACM2; Kruse et al., 2013)  Nb39 (OPRM; Huang et al., 2015)  Nb39 (OPRK; Che et al., 2020)  Nb.AT110 and AT110i1 (AGTR1; Wingler et al., 2019)  **Type II**  Nb60 (ADRB2; Staus et al., 2016)  Nb6 (OPRK; Che et al., 2020)  **Type III**  VGS-Nb2 (5HT2A:Gq; English et al., 2019) |
| I. Modulation of ligand affinity to GPCR by Cb | *Radioligand - saturation.* This assay uses agonist radioligand as key reagent. It measures maximum amount of binding sites (Bmax) of the agonist radioligand modulated by conformer-stabilizing VHHs. The ligand’s affinity constant (Kd) can be calculated from the Bmax value. A VHH that causes a significant Bmax increase or decrease compared to the condition using apo receptor is an active or inactive conformer-stabilizing VHH, respectively. As a variation of the assay, the impact of the conformer-stabilizing VHH (compared to the impact of an irrelevant VHH) on a single agonist radioligand concentration to the receptor can be measured. | **Type I**  Nb80 (ADRB2; Staus et al.,2014)  Nb39 (OPRK; Che et al., 2018 and 2020)  **Type II**  Nb60 (ADRB2; Staus et al.,2014)  Nb6 (OPRK; Che et al., 2020)  **Type III**  VGS-Nb2 (5HT2A:Gq; English et al., 2019) |
| II. Modulation of Cb binding to GPCR by ligand | *Flow cytometry – VHH displayed on yeast cell.* The assay measures binding of yeast cell surface expressed VHH to ligand occupied fluorescently labeled purified receptor providing MCF (mean/median cell fluorescence) values. A VHH is active conformer-stabilizing when it preferentially binds to agonist occupied GPCR versus antagonist doped GPCR.  *Flow cytometry – GPCR overexpressed on eukaryotic cell.* An alternative set-up measures binding of (indirectly labeled) fluorescent VHH to insect cells over expressing the receptor of interest. By default, a partial population of Sf9 cells becomes permeable following baculovirus infection (allowing VHHs to penetrate cells). Different conformer-stabilizing VHH profiles of antibodies can be discriminated by comparing binding events in the presence of excess agonist versus antagonist or no ligand bound receptor expressing cells. Gating with a cell viability dye enables to define whether the VHH interacts with extracellulular or intracellular epitope as VHH are not able to stain intracellular epitopes of the receptor on intact viable cells. | **Type I**  Nb6B9 (ADRB2; Ring et al., 2013)  Nb.c200 (ADRB2; McMahon et al.; 2018)  Nb.AD101 and Nb.AD102 (AA2AR; McMahon et al., 2018)  Nb9-8 (ACM2; Kruse et al., 2013)  Nb.AT110 (AGTR1; Wingler et al., 2019)  NbSmo8 (SMO; Deshpande et al., 2019)  **Type I** Nb80 and **Type II** Nb60 (ADRB2; Staus et al., 2014) |
| II. Modulation of Cb binding to GPCR by ligand | *Enzyme linked immunosorbent assay.* ELISA is used to characterize the binding propensity of the GPCR to immobilized VHH. VHH captured purified detergent-soluble receptor (agonist or inverse agonist occupied) is detected by a detection antibody to receptor tag. The selective binding of VHH to agonist or inverse agonist doped receptor indicates preferential binding of VHH to active state receptor or inactive state conformers. | **Type I** Nb80 and **Type II** Nb60 (ADRB2; Staus et al., 2014) |
| II. Modulation of Cb binding to GPCR by ligand | *Affinity enriched complex (pull-down,* immunoprecipitation, size exclusion chromatography).  Co-purification of conformer-stabilizing VHHs and ligand occupied detergent soluble receptor (or prevention of GTPγgS induced dissociation of ternary GPCR:VHH complex with transducer) via size exclusion chromatography demonstrates stable complex formation in the presence of agonist prior to crystallization studies. Complex formation can also be assessed after pull-down comparing binding to agonist and antagonist occupied receptor preps (using an antibody to the tagged receptor) and subsequent SDS-PAGE. Yet another approach is to assess complex formation via immunoblot. Following ligand stimulation of mammalian cells co-transfected with GPCR and VHH, GPCR-VHH complex is detected via immune precipitation. For all above-described complex formation assays, a VHH that preferentially binds to the receptor or receptor transducer complex in presence of agonist is an indicator of an active state conformer-stabilizing VHH. | **Type I**  Nb80 (ADRB2; Staus et al.,2014)  Nb.AD101 and Nb.AD102 (AA2AR; McMahon et al., 2018)  Nb.AT110 (AGTR1; Wingler et al. 2019)  NbSmo8 (SMO; Deshpande et al., 2019)  **Type II**  Nb60 (ADRB2; Staus et al.,2014)  **Type III**  Nb35 (ADRB2:Gs; Rasmussen et al., 2011b) |
| II. Modulation of Cb binding to GPCR by ligand | *Surface plasmon resonance and biolayer interferometry.*  Comparative binding of agonist or antagonist occupied GPCR to immobilized VHH is assessed. An active state stabilizing VHH binds preferentially to agonist occupied while not to antagonist doped receptor. | **Type I**  NbSmo8 (SMO; Deshpande et al., 2019)  Nb39 (OPRM; Livingston et al., 2018) |
| III. Biosensor – ligand selective recruitment of VHH to GPCR | *Ligand induced recruitment of G protein mimic.* The conformational change of the GPCR consequent to agonist binding translocates distant cytosolic signaling transducers such as G proteins (or G protein mimicking VHHs) to the receptor’s vicinity. Upon co-transfection of a mammalian host, GPCR and VHH co-localization is monitored via bioluminescence resonance energy transfer (BRET), split luciferase or total internal reflection fluorescence microscopy (TIRFM). A VHH that preferentially translocates to the receptor’s vicinity (or receptor transducer complex) in presence of agonist but not antagonist or inverse agonist or in absence of ligand is an indication of an active state conformer-stabilizing VHH. Inversely, a VHH that preferentially translocates to the receptor’s vicinity in presence of antagonist or inverse agonist is an indication of an inactive state conformer-stabilizing VHH. | **Type I**  Nb80 (ADRB2 ; Irranejad et al., 2013)  Nb39 (OPRM; Stoeber et al. 2018 and Vasudevan and Stove 2020)  Nb39 (OPRK; Che et al., 2018 and 2020)  **Type II**  Nb6 (OPRK; Che et al., 2018 and 2020)  **Type III**  Nb37 (ADRB2:Gs; Irranejad et al., 2013)  VGS-Nb2 (5HT2A:Gq; English et al., 2019) |
| IV. Transmembrane α-helical movement | *Fluorescence emission assay.* Different conformational states can be distinguished by tracking movement of TM6 using a monobromobimane probe attached to the cytoplasmic end of TM6. During receptor activation, the cooperative binding of agonist ligand and G protein (or G protein mimicking VHH) induces a transmembrane α-helix 6 (TM6) outward movement. Such TM6 movement causes the exposure of the bimane probe, resulting in a decrease of detected fluorescence. A VHH that in presence of an agonist (but not in presence of an antagonist or inverse agonist) shows a cooperative decrease in fluorescence is an indicator of an active state conformer-stabilizing VHH. | **Type I**  Nb80 (ADRB2; Rasmussen et al. 2011a)  Nb80/Nb71 (ADRB2; Masureel et al., 2018) |

**Supplementary Table S3.** Overview and specifics of ConfoBody enabled protein structures in PDB. For uniformity in GPCR nomenclature in this review, the GPCR synonym of the Uniprot database chosen is the one indicated in Fig. 4. Abbreviations: X-ray diffraction (X-ray), electron microscopy (EM).

| **PDB code** | **GPCR Uniprot ID** | **GPCR Uniprot Gene Name** | **Method used to solve structure** | **Resolution (Å)** | **GPCR class** | **State (A=active state, I=inactive state)** | **ConfoBody directly associated with** | **Cb Identifier** | **PDB date** | **Reference** |
| --- | --- | --- | --- | --- | --- | --- | --- | --- | --- | --- |
| 5VAI | G1SGD4 | GLP1R | EM | 4.1 | B | A | G protein complexed with GPCR | Nb35 | 27/03/2017 | 10.1038/nature22394 |
| 7DH5 | O02662 | ADRB3 | EM | 3.2 | A | A | G protein complexed with GPCR | Nb35 | 04/08/2021 | 10.1016/J.MOLCEL.2021.06.024 |
| 7EVW | O75084 | FZD7 | EM | 3.2 | F | A | G protein complexed with GPCR | Nb35 | 04/08/2021 | 10.1038/S41422-021-00525-6 |
| 7D68 | O95838 | GLP2R | EM | 3.0 | B | A | G protein complexed with GPCR | Nb35 | 16/12/2020 | 10.1038/S41422-020-00442-0 |
| 6OYA | P02699 | OPSD | EM | 3.3 | A | A | G protein complexed with GPCR | Nb35 | 14/05/2019 | 10.1016/j.molcel.2019.06.007 |
| 7BZ2 | P07550 | ADRB2 | EM | 3.8 | A | A | G protein complexed with GPCR | Nb35 | 05/08/2020 | 10.1038/S41421-020-0176-9 |
| 7DHI | P07550 | ADRB2 | EM | 3.3 | A | A | G protein complexed with GPCR | Nb35 | 16/12/2020 | 10.1093/NSR/NWAA284 |
| 7DHR | P07550 | ADRB2 | EM | 3.8 | A | A | G protein complexed with GPCR | Nb35 | 16/12/2020 | 10.1093/NSR/NWAA284 |
| 3SN6 | P07550 | ADRB2 | X-RAY | 3.2 | A | A | G protein complexed with GPCR | Nb35 | 28/06/2011 | 10.1038/nature10361 |
| 6NI3 | P07550 | ADRB2 | EM | 3.8 | A | A | G protein complexed with GPCR | Nb35 | 26/12/2018 | 10.1038/s41594-019-0330-y |
| 5JQH | P07550 | ADRB2 | X-RAY | 3.2 | A | I | GPCR on IC aspect | Nb60 | 05/05/2016 | 10.1038/nature18636 |
| 4LDE | P07550 | ADRB2 | X-RAY | 2.8 | A | A | GPCR on IC aspect | Nb6B9 | 24/06/2013 | 10.1038/nature12572 |
| 4LDL | P07550 | ADRB2 | X-RAY | 3.1 | A | A | GPCR on IC aspect | Nb6B9 | 24/06/2013 | 10.1038/nature12572 |
| 4LDO | P07550 | ADRB2 | X-RAY | 3.2 | A | A | GPCR on IC aspect | Nb6B9 | 24/06/2013 | 10.1038/nature12572 |
| 4QKX | P07550 | ADRB2 | X-RAY | 3.3 | A | A | GPCR on IC aspect | Nb6B9 | 10/06/2014 | 10.1073/pnas.1410415111 |
| 6N48 | P07550 | ADRB2 | X-RAY | 3.2 | A | A | GPCR on IC aspect | Nb6B9 | 17/11/2018 | 10.1126/science.aaw8981 |
| 6MXT | P07550 | ADRB2 | X-RAY | 3.0 | A | A | GPCR on IC aspect | Nb71 | 31/10/2018 | 10.1038/s41589-018-0145-x |
| 3P0G | P07550 | ADRB2 | X-RAY | 3.5 | A | A | GPCR on IC aspect | Nb80 | 28/09/2010 | 10.1038/nature09648 |
| 7JJO | P07700 | ADRB1 | EM | 2.6 | A | A | G protein complexed with GPCR | Nb35 | 02/09/2020 | 10.1016/J.MOLCEL.2020.08.001 |
| 6H7L | P07700 | ADRB1 | X-RAY | 2.7 | A | A | GPCR on IC aspect | Nb6B9 | 31/07/2018 | 10.1126/science.aau5595 |
| 6H7M | P07700 | ADRB1 | X-RAY | 2.8 | A | A | GPCR on IC aspect | Nb6B9 | 31/07/2018 | 10.1126/science.aau5595 |
| 6H7N | P07700 | ADRB1 | X-RAY | 2.5 | A | A | GPCR on IC aspect | Nb6B9 | 31/07/2018 | 10.1126/science.aau5595 |
| 6H7O | P07700 | ADRB1 | X-RAY | 2.8 | A | A | GPCR on IC aspect | Nb6B9 | 31/07/2018 | 10.1126/science.aau5595 |
| 6H7J | P07700 | ADRB1 | X-RAY | 2.8 | A | A | GPCR on IC aspect | Nb80 | 31/07/2018 | 10.1126/science.aau5595 |
| 6IBL | P07700 | ADRB1 | X-RAY | 2.7 | A | A | GPCR on IC aspect | Nb80 | 30/11/2018 | 10.1038/s41586-020-2419-1 |
| 4MQS | P08172 | ACM2 | X-RAY | 3.5 | A | A | GPCR on IC aspect | Nb9-8 | 16/09/2013 | 10.1038/nature12735 |
| 4MQT | P08172 | ACM2 | X-RAY | 3.7 | A | A | GPCR on IC aspect | Nb9-8 | 16/09/2013 | 10.1038/nature12735 |
| 7BTS | P08588 | ADRB1 | X-RAY | 3.1 | A | A | GPCR on IC aspect | Nb6B9 | 02/12/2020 | 10.1038/S41422-020-00424-2 |
| 7BU6 | P08588 | ADRB1 | X-RAY | 2.7 | A | A | GPCR on IC aspect | Nb6B9 | 02/12/2020 | 10.1038/S41422-020-00424-2 |
| 7BU7 | P08588 | ADRB1 | X-RAY | 2.6 | A | A | GPCR on IC aspect | Nb6B9 | 02/12/2020 | 10.1038/S41422-020-00424-2 |
| 7CKW | P21728 | DRD1 | EM | 3.2 | A | A | G protein complexed with GPCR | Nb35 | 03/03/2021 | 10.1016/J.CELL.2021.01.028 |
| 7CKX | P21728 | DRD1 | EM | 3.5 | A | A | G protein complexed with GPCR | Nb35 | 03/03/2021 | 10.1016/J.CELL.2021.01.028 |
| 7CKY | P21728 | DRD1 | EM | 3.2 | A | A | G protein complexed with GPCR | Nb35 | 03/03/2021 | 10.1016/J.CELL.2021.01.028 |
| 7CKZ | P21728 | DRD1 | EM | 3.1 | A | A | G protein complexed with GPCR | Nb35 | 03/03/2021 | 10.1016/J.CELL.2021.01.028 |
| 7CRH | P21728 | DRD1 | EM | 3.3 | A | A | G protein complexed with GPCR | Nb35 | 03/03/2021 | 10.1016/J.CELL.2021.01.028 |
| 7JOZ | P21728 | DRD1 | X-RAY | 3.8 | A | A | G protein complexed with GPCR | Nb35 | 14/04/2021 | 10.1038/s41467-021-23519-9 |
| 7JV5 | P21728 | DRD1 | EM | 3.0 | A | A | G protein complexed with GPCR | Nb35 | 24/02/2021 | 10.1016/J.CELL.2021.01.027 |
| 7JVP | P21728 | DRD1 | EM | 2.9 | A | A | G protein complexed with GPCR | Nb35 | 24/02/2021 | 10.1016/J.CELL.2021.01.027 |
| 7JVQ | P21728 | DRD1 | EM | 3.0 | A | A | G protein complexed with GPCR | Nb35 | 24/02/2021 | 10.1016/J.CELL.2021.01.027 |
| 7LJC | P21728 | DRD1 | EM | 3.0 | A | A | G protein complexed with GPCR | Nb35 | 03/03/2021 | 10.1038/s41422-021-00482-0 |
| 7LJD | P21728 | DRD1 | EM | 3.2 | A | A | G protein complexed with GPCR | Nb35 | 03/03/2021 | 10.1038/s41422-021-00482-0 |
| 7FIG | P22888 | LSHR | EM | 3.9 | A | A | G protein complexed with GPCR | Nb35 | 29/09/2021 | 10.1038/S41586-021-03924-2 |
| 6GDG | P29274 | AA2AR | EM | 4.1 | A | A | G protein complexed with GPCR | Nb35 | 23/04/2018 | 10.7554/eLife.35946 |
| 7BB6 | P30518 | V2R | EM | 4.2 | A | A | G protein complexed with GPCR | Nb35 | 02/06/2021 | 10.1126/SCIADV.ABG5628 |
| 7BB7 | P30518 | V2R | EM | 4.4 | A | A | G protein complexed with GPCR | Nb35 | 02/06/2021 | 10.1126/SCIADV.ABG5628 |
| 7DW9 | P30518 | V2R | EM | 2.6 | A | A | G protein complexed with GPCR | Nb35 | 21/07/2021 | 10.1038/S41422-021-00480-2 |
| 7KH0 | P30518 | V2R | EM | 2.8 | A | A | G protein complexed with GPCR | Nb35 | 26/05/2021 | 10.1038/S41422-021-00483-Z |
| 6DO1 | P30556 | AGTR1 | X-RAY | 2.9 | A | A | GPCR on IC aspect | Nb.AT110i1 | 08/06/2018 | 10.1016/j.cell.2018.12.006 |
| 6OS0 | P30556 | AGTR1 | X-RAY | 2.9 | A | A | GPCR on IC aspect | Nb.AT110i1 | 01/05/2019 | 10.1126/science.aay9813 |
| 6OS1 | P30556 | AGTR1 | X-RAY | 2.8 | A | A | GPCR on IC aspect | Nb.AT110i1 | 01/05/2019 | 10.1126/science.aay9813 |
| 6OS2 | P30556 | AGTR1 | X-RAY | 2.7 | A | A | GPCR on IC aspect | Nb.AT110i1 | 01/05/2019 | 10.1126/science.aay9813 |
| 5UZ7 | P30988 | CALCR | EM | 4.1 | B | A | G protein complexed with GPCR | Nb35 | 24/02/2017 | 10.1038/nature22327 |
| 6NIY | P30988 | CALCR | EM | 3.3 | B | A | G protein complexed with GPCR | Nb35 | 02/01/2019 | 10.1021/acsptsci.8b00056 |
| 7MBX | P32238 | CCKAR | EM | 2.0 | A | A | G protein complexed with GPCR | Nb35 | 26/05/2021 | 10.1371/journal.pbio.3001295 |
| 6VN7 | P32241 | VIPR1 | EM | 3.2 | B | A | G protein complexed with GPCR | Nb35 | 02/09/2020 | 10.1038/S41467-020-17933-8 |
| 7F53 | P32245 | MC4R | EM | 3.0 | A | A | G protein complexed with GPCR | Nb35 | 03/11/2021 | 10.1038/s41422-021-00552-3 |
| 7F54 | P32245 | MC4R | EM | 3.0 | A | A | G protein complexed with GPCR | Nb35 | 03/11/2021 | 10.1038/s41422-021-00552-3 |
| 7F55 | P32245 | MC4R | EM | 3.1 | A | A | G protein complexed with GPCR | Nb35 | 03/11/2021 | 10.1038/s41422-021-00552-3 |
| 7F58 | P32245 | MC4R | EM | 3.1 | A | A | G protein complexed with GPCR | Nb35 | 03/11/2021 | 10.1038/s41422-021-00552-3 |
| 7PIU | P32245 | MC4R | EM | 2.6 | A | A | G protein complexed with GPCR | Nb35 | 17/11/2021 | 10.1038/s41422-021-00569-8 |
| 7PIV | P32245 | MC4R | EM | 2.9 | A | A | G protein complexed with GPCR | Nb35 | 17/11/2021 | 10.1038/s41422-021-00569-8 |
| 6P9X | P34998 | CRHR1 | EM | 2.9 | B | A | G protein complexed with GPCR | Nb35 | 10/06/2019 | 10.1016/j.molcel.2020.01.012 |
| 6PB0 | P34998 | CRHR1 | EM | 3.0 | B | A | G protein complexed with GPCR | Nb35 | 12/06/2019 | 10.1016/j.molcel.2020.01.013 |
| 7D7M | P35408 | PE2R4 | EM | 3.3 | A | A | G protein complexed with GPCR | Nb35 | 18/11/2020 | 10.1016/j.str.2020.11.007 |
| 6B73 | P41145 | OPRK | X-RAY | 3.1 | A | A | GPCR on IC aspect | Nb39 | 03/10/2017 | 10.1016/j.cell.2017.12.011 |
| 6VI4 | P41145 | OPRK | X-RAY | 3.3 | A | I | GPCR on IC aspect | Nb6 | 11/01/2020 | 10.1038/s41467-020-14889-7 |
| 6LPB | P41586 | PACR | EM | 3.9 | B | A | G protein complexed with GPCR | Nb35 | 09/01/2020 | 10.1038/s41594-020-0386-8 |
| 6P9Y | P41586 | PACR | EM | 3.0 | B | A | G protein complexed with GPCR | Nb35 | 10/06/2019 | 10.1016/j.molcel.2020.01.012 |
| 5C1M | P42866 | OPRM | X-RAY | 2.1 | A | A | GPCR on IC aspect | Nb39 | 15/06/2015 | 10.1038/nature14886 |
| 7CX2 | P43116 | PE2R2 | EM | 2.8 | A | A | G protein complexed with GPCR | Nb35 | 05/05/2021 | 10.1126/SCIADV.ABF1268 |
| 7CX3 | P43116 | PE2R2 | EM | 2.8 | A | A | G protein complexed with GPCR | Nb35 | 05/05/2021 | 10.1126/SCIADV.ABF1268 |
| 7CX4 | P43116 | PE2R2 | EM | 2.9 | A | A | G protein complexed with GPCR | Nb35 | 05/05/2021 | 10.1126/SCIADV.ABF1268 |
| 6VCB | P43220 | GLP1R | EM | 3.3 | B | A | G protein complexed with GPCR | Nb35 | 22/07/2020 | 10.1038/S41589-020-0589-7 |
| 6X18 | P43220 | GLP1R | EM | 2.1 | B | A | G protein complexed with GPCR | Nb35 | 09/09/2020 | 10.1101/2020.08.16.252585 |
| 6X19 | P43220 | GLP1R | EM | 2.1 | B | A | G protein complexed with GPCR | Nb35 | 09/09/2020 | 10.1101/2020.08.16.252585 |
| 6X1A | P43220 | GLP1R | EM | 2.5 | B | A | G protein complexed with GPCR | Nb35 | 09/09/2020 | 10.1101/2020.08.16.252585 |
| 6XOX | P43220 | GLP1R | EM | 3.1 | B | A | G protein complexed with GPCR | Nb35 | 18/11/2020 | 10.1073/PNAS.2014879117 |
| 7C2E | P43220 | GLP1R | EM | 4.2 | B | A | G protein complexed with GPCR | Nb35 | 26/08/2020 | 10.1038/S41422-020-0384-8 |
| 7DUQ | P43220 | GLP1R | EM | 2.5 | B | A | G protein complexed with GPCR | Nb35 | 14/07/2021 | 10.1038/S41467-021-24058-Z |
| 7DUR | P43220 | GLP1R | EM | 3.3 | B | A | G protein complexed with GPCR | Nb35 | 11/08/2021 | 10.1038/S41467-021-24058-Z |
| 7E14 | P43220 | GLP1R | EM | 2.9 | B | A | G protein complexed with GPCR | Nb35 | 07/07/2021 | 10.1038/S41467-021-24058-Z |
| 7EVM | P43220 | GLP1R | EM | 2.5 | B | A | G protein complexed with GPCR | Nb35 | 11/08/2021 | 10.1038/S41467-021-24058-Z |
| 7KI0 | P43220 | GLP1R | EM | 2.5 | B | A | G protein complexed with GPCR | Nb35 | 04/08/2021 | 10.1016/J.CELREP.2021.109374 |
| 7KI1 | P43220 | GLP1R | EM | 2.5 | B | A | G protein complexed with GPCR | Nb35 | 04/08/2021 | 10.1016/J.CELREP.2021.109374 |
| 6B3J | P43220 | GLP1R | EM | 3.3 | B | A | G protein complexed with GPCR | Nb35 | 22/09/2017 | 10.1038/nature25773 |
| 6ORV | P43220 | GLP1R | EM | 3.0 | B | A | G protein complexed with GPCR | Nb35 | 01/05/2019 | 10.1038/s41586-019-1902-z |
| 6WHC | P47871 | GCGR | EM | 3.4 | B | A | G protein complexed with GPCR | Nb35 | 27/05/2020 | 10.1074/JBC.RA120.013793 |
| 6WPW | P47871 | GCGR | EM | 3.1 | B | A | G protein complexed with GPCR | Nb35 | 12/08/2020 | 10.1126/SCIENCE.ABA3373 |
| 6LMK | P47871 | GCGR | EM | 3.7 | B | A | G protein complexed with GPCR | Nb35 | 26/12/2019 | 10.1126/science.aaz5346 |
| 6WI9 | P47872 | SCTR | EM | 4.3 | B | A | G protein complexed with GPCR | Nb35 | 12/08/2020 | 10.1038/S41467-020-17791-4 |
| 6WZG | P47872 | SCTR | EM | 2.3 | B | A | G protein complexed with GPCR | Nb35 | 12/08/2020 | 10.1038/S41467-020-17791-4 |
| 7D3S | P47872 | SCTR | EM | 2.9 | B | A | G protein complexed with GPCR | Nb35 | 04/11/2020 | 10.1016/J.BBRC.2020.08.042 |
| 7DTY | P48546 | GIPR | EM | 3.0 | B | A | G protein complexed with GPCR | Nb35 | 04/08/2021 | 10.7554/ELIFE.68719 |
| 7F16 | P49190 | PTH2R | EM | 2.8 | B | A | G protein complexed with GPCR | Nb35 | 18/08/2021 | 10.1073/PNAS.2101279118 |
| 6O3C | P56726 | SMO | X-RAY | 2.8 | F | A | GPCR on IC aspect | NbSmo8 | 26/02/2019 | 10.1038/s41586-019-1355-4 |
| 4XT1 | P69332 | US28 | X-RAY | 2.9 | A | A | GPCR on IC aspect | Nb7 | 22/01/2015 | 10.1126/science.aaa5026 |
| 5WB1 | P69332 | US28 | X-RAY | 3.5 | A | A | GPCR on IC aspect | Nb7 | 27/06/2017 | 10.7554/eLife.35850 |
| 5WB2 | P69332 | US28 | X-RAY | 3.5 | A | A | GPCR on IC aspect | Nb7 | 27/06/2017 | 10.7554/eLife.35850 |
| 7CZ5 | Q02643 | GHRHR | EM | 2.6 | B | A | G protein complexed with GPCR | Nb35 | 18/11/2020 | 10.1038/S41467-020-18945-0 |
| 6NBF | Q03431 | PTH1R | EM | 3.0 | B | A | G protein complexed with GPCR | Nb35 | 07/12/2018 | 10.1126/science.aav7942 |
| 6NBH | Q03431 | PTH1R | EM | 3.5 | B | A | G protein complexed with GPCR | Nb35 | 07/12/2018 | 10.1126/science.aav7942 |
| 6NBI | Q03431 | PTH1R | EM | 4.0 | B | A | G protein complexed with GPCR | Nb35 | 07/12/2018 | 10.1126/science.aav7942 |
| 6PB1 | Q13324 | CRHR2 | EM | 2.8 | B | A | G protein complexed with GPCR | Nb35 | 12/06/2019 | 10.1016/j.molcel.2020.01.013 |
| 6UVA | Q16602 | CALCRL | EM | 2.3 | B | A | G protein complexed with GPCR | Nb35 | 01/04/2020 | 10.1021/ACSPTSCI.9B00080 |
| 6E3Y | Q16602 | CALCRL | EM | 3.3 | B | A | G protein complexed with GPCR | Nb35 | 16/07/2018 | 10.1038/s41586-018-0535-y |
| 6UUN | Q16602 | CALCRL | EM | 3.0 | B | A | G protein complexed with GPCR | Nb35 | 30/10/2019 | 10.1021/acsptsci.9b00080 |
| 6UUS | Q16602 | CALCRL | EM | 2.4 | B | A | G protein complexed with GPCR | Nb35 | 31/10/2019 | 10.1021/acsptsci.9b00080 |
| 6Z10 | Q6IYF9 | SUCR1 | X-RAY | 2.3 | A | I | GPCR on IC aspect | Nanobody 6 | 16/09/2020 | 10.1021/ACS.JMEDCHEM.0C01020 |
| 6IBB | Q6IYF9 | SUCR1 | X-RAY | 2.1 | A | I | GPCR on IC aspect | Nanobody 6 | 29/11/2018 | 10.1038/s41586-019-1663-8 |
| 6RNK | Q6IYF9 | SUCR1 | X-RAY | 1.9 | A | I | GPCR on IC aspect | Nanobody 6 | 08/05/2019 | 10.1038/s41586-019-1663-8 |
| 7BW0 | Q8TDU6 | GPBAR | EM | 3.9 | A | A | G protein complexed with GPCR | Nb35 | 02/09/2020 | 10.1038/S41392-020-00262-Z |
| 7CFM | Q8TDU6 | GPBAR | EM | 3.0 | A | A | G protein complexed with GPCR | Nb35 | 09/09/2020 | 10.1038/S41586-020-2569-1 |
| 7CFN | Q8TDU6 | GPBAR | EM | 3.0 | A | A | G protein complexed with GPCR | Nb35 | 09/09/2020 | 10.1038/S41586-020-2569-1 |
| 6LI3 | Q9Y2T5 | GPR52 | EM | 3.3 | A | A | G protein complexed with GPCR | Nb35 | 10/12/2019 | 10.1038/s41586-020-2019-0 |

**Supplementary Table S4.** Cb enabled GPCR structures in PDB database. For uniformity in GPCR nomenclature in this review, the GPCR synonym of the Uniprot database chosen is the one indicated in Fig. 4.

| **GPCR Uniprot ID** | **Nanobody 6^ii^** | **Nb.AT110i1^i^** | **Nb35^iii^** | **Nb39^i^** | **Nb6^ii^** | **Nb60^ii^** | **Nb6B9^i^** | **Nb7^i^** | **Nb71^i^** | **Nb80^i^** | **Nb9-8^i^** | **NbSmo8^i^** | **Grand Total** |
| --- | --- | --- | --- | --- | --- | --- | --- | --- | --- | --- | --- | --- | --- |
| AA2AR |  |  | 1 |  |  |  |  |  |  |  |  |  | 1 |
| ADRB1 |  |  | 1 |  |  |  | 7 |  |  | 2 |  |  | 10 |
| ADRB2 |  |  | 5 |  |  | 1 | 5 |  | 1 | 1 |  |  | 13 |
| ADRB3 |  |  | 1 |  |  |  |  |  |  |  |  |  | 1 |
| AGTR1 |  | 4 |  |  |  |  |  |  |  |  |  |  | 4 |
| V2R |  |  | 4 |  |  |  |  |  |  |  |  |  | 4 |
| CALCR |  |  | 2 |  |  |  |  |  |  |  |  |  | 2 |
| CALCRL |  |  | 4 |  |  |  |  |  |  |  |  |  | 4 |
| CCKAR |  |  | 1 |  |  |  |  |  |  |  |  |  | 1 |
| ACM2 |  |  |  |  |  |  |  |  |  |  | 2 |  | 2 |
| CRHR1 |  |  | 2 |  |  |  |  |  |  |  |  |  | 2 |
| CRHR2 |  |  | 1 |  |  |  |  |  |  |  |  |  | 1 |
| DRD1 |  |  | 11 |  |  |  |  |  |  |  |  |  | 11 |
| FZD7 |  |  | 1 |  |  |  |  |  |  |  |  |  | 1 |
| GCGR |  |  | 3 |  |  |  |  |  |  |  |  |  | 3 |
| GHRHR |  |  | 1 |  |  |  |  |  |  |  |  |  | 1 |
| GIPR |  |  | 1 |  |  |  |  |  |  |  |  |  | 1 |
| GLP1R |  |  | 15 |  |  |  |  |  |  |  |  |  | 15 |
| GLP2R |  |  | 1 |  |  |  |  |  |  |  |  |  | 1 |
| GPBAR |  |  | 3 |  |  |  |  |  |  |  |  |  | 3 |
| GPR52 |  |  | 1 |  |  |  |  |  |  |  |  |  | 1 |
| LSHR |  |  | 1 |  |  |  |  |  |  |  |  |  | 1 |
| MC4R |  |  | 6 |  |  |  |  |  |  |  |  |  | 6 |
| OPRK |  |  |  | 1 | 1 |  |  |  |  |  |  |  | 2 |
| OPRM |  |  |  | 1 |  |  |  |  |  |  |  |  | 1 |
| PACR |  |  | 2 |  |  |  |  |  |  |  |  |  | 2 |
| PE2R2 |  |  | 3 |  |  |  |  |  |  |  |  |  | 3 |
| PE2R4 |  |  | 1 |  |  |  |  |  |  |  |  |  | 1 |
| PTH1R |  |  | 3 |  |  |  |  |  |  |  |  |  | 3 |
| PTH2R |  |  | 1 |  |  |  |  |  |  |  |  |  | 1 |
| OPSD |  |  | 1 |  |  |  |  |  |  |  |  |  | 1 |
| SCTR |  |  | 3 |  |  |  |  |  |  |  |  |  | 3 |
| SMO |  |  |  |  |  |  |  |  |  |  |  | 1 | 1 |
| SUCR1 | 3 |  |  |  |  |  |  |  |  |  |  |  | 3 |
| US28 |  |  |  |  |  |  |  | 3 |  |  |  |  | 3 |
| VIPR1 |  |  | 1 |  |  |  |  |  |  |  |  |  | 1 |
| **Grand Total** | **3** | **4** | **81** | **2** | **1** | **1** | **12** | **3** | **1** | **3** | **2** | **1** | **114** |

^i^Type I Cb

^ii^Type II Cb

^iii^Type III Cb

**Supplementary references**

Burg, J.S., Ingram, J.R., Venkatakrishnan, A.J., Jude, K.M., Dukkipati, A., Feinberg, E.N., Angelini, A., Waghray, D., Dror, R.O., Ploegh, H.L., and Garcia, K.C. (2015). Structural basis for chemokine recognition and activation of a viral G protein-coupled receptor. Science 347, 1113-1117. doi: 10.1126/science.aaa5026

Cahill, T.J. 3rd, Thomsen, A.R., Tarrasch, J.T., Plouffe, B., Nguyen, A.H., Yang, F., Huang, L.Y., Kahsai, A.W., Bassoni, D.L., Gavino, B.J., Lamerdin, J.E., Triest, S., Shukla, A.K., Berger, B., Little, J. 4th, Antar, A., Blanc, A., Qu, C.X., Chen, X., Kawakami, K., Inoue, A., Aoki, J., Steyaert, J., Sun, J.P., Bouvier, M., Skiniotis, G., and Lefkowitz, R.J. (2017). Distinct conformations of GPCR-β-arrestin complexes mediate desensitization, signaling, and endocytosis. Proc. Natl. Acad. Sci. USA. 114, 2562-2567. doi: 10.1073/pnas.1701529114

Che, T., Majumdar, S., Zaidi, S.A., Ondachi, P., McCorvy, J.D., Wang, S., Mosier, P.D., Uprety, R., Vardy, E., Krumm, B.E., Han, G.W., Lee, M.Y., Pardon, E., Steyaert, J., Huang, X.P., Strachan, R.T., Tribo, A.R., Pasternak, G.W., Carroll, F.I., Stevens, R.C., Cherezov, V., Katritch, V., Wacker, D., and Roth, B.L. (2018). Structure of the Nanobody-Stabilized Active State of the Kappa Opioid Receptor. Cell 172, 55-67. doi: 10.1016/j.cell.2017.12.011.

Che, T., English, J., Krumm, B.E., Kim, K., Pardon, E., Olsen, R.H.J., Wang, S., Zhang, S., Diberto, J.F., Sciaky, N., Carroll, F.I., Steyaert, J., Wacker, D., and Roth, B.L. (2020). Nanobody-enabled monitoring of kappa opioid receptor states. Nat. Commun. 11, 1145. doi: 10.1038/s41467-020-14889-7

De Groof, T.W.M., Bergkamp, N.D., Heukers, R., Giap, T., Bebelman, M.P., Goeij-de Haas, R., Piersma, S.R., Jimenez, C.R., Garcia, K.C., Ploegh, H.L., Siderius, M., and Smit, M.J. (2021). Selective targeting of ligand-dependent and -independent signaling by GPCR conformation-specific anti-US28 intrabodies. Nat. Commun. 12, 4357. doi: 10.1038/s41467-021-24574-y

Deshpande, I., Liang, J., Hedeen, D., Roberts, K. J., Zhang, Y., Ha, B., Latorraca, N. R., Faust, B., Dror, R. O., Beachy, P. A., Myers, B. R., & Manglik, A. (2019). Smoothened stimulation by membrane sterols drives Hedgehog pathway activity. Nature, 571, 284–288. doi.org/10.1038/s41586-019-1355-4

DeVree, B. T., Mahoney, J. P., Vélez-Ruiz, G. A., Rasmussen, S. G., Kuszak, A. J., Edwald, E., Fung, J. J., Manglik, A., Masureel, M., Du, Y., Matt, R. A., Pardon, E., Steyaert, J., Kobilka, B. K., and Sunahara, R. K. (2016). Allosteric coupling from G protein to the agonist-binding pocket in GPCRs. Nature, 535, 182–186. doi.org/10.1038/nature18324

English JG, Olsen RHJ, Lansu K, Patel M, White K, Cockrell AS, Singh D, Strachan RT, Wacker D, and Roth BL (2019). VEGAS as a Platform for Facile Directed Evolution in Mammalian Cells. Cell 178, 748-761. doi: 10.1016/j.cell.2019.05.051

Haffke, M., Fehlmann, D., Rummel, G., Boivineau, J., Duckely, M., Gommermann, N., Cotesta, S., Sirockin, F., Freuler, F., Littlewood-Evans, A., Kaupmann, K., and Jaakola, V. P. (2019). Structural basis of species-selective antagonist binding to the succinate receptor. Nature, 574, 581–585. doi.org/10.1038/s41586-019-1663-8

Huang, W., Manglik, A., Venkatakrishnan, A.J., Laeremans, T., Feinberg, E.N., Sanborn, A.L., Kato, H.E., Livingston, K.E., Thorsen, T.S., Kling, R.C., Granier, S., Gmeiner, P., Husbands, S.M., Traynor, J.R., Weis, W.I., Steyaert, J., Dror, R.O., and Kobilka B.K (2015). Structural insights into µ-opioid receptor activation. Nature 524, 315-321. doi: 10.1038/nature14886

Irannejad, R., Tomshine, J. C., Tomshine, J. R., Chevalier, M., Mahoney, J. P., Steyaert, J., Rasmussen, S. G., Sunahara, R. K., El-Samad, H., Huang, B., and von Zastrow, M. (2013). Conformational biosensors reveal GPCR signalling from endosomes. Nature, 495, 534–538. doi.org/10.1038/nature12000

Kruse, A.C., Ring, A.M., Manglik, A., Hu, J., Hu, K., Eitel, K., Hübner, H., Pardon, E., Valant, C., Sexton, P.M., Christopoulos, A., Felder, C.C., Gmeiner, P., Steyaert, J., Weis, W.I., Garcia, K.C., Wess, J., and Kobilka, B.K. (2013). Activation and allosteric modulation of a muscarinic acetylcholine receptor. Nature 504, 101-106. doi: 10.1038/nature12735

Liu X, Masoudi A, Kahsai AW, Huang LY, Pani B, Staus DP, Shim PJ, Hirata K, Simhal RK, Schwalb AM, Rambarat PK, Ahn S, Lefkowitz RJ, Kobilka B. Mechanism of β2AR regulation by an intracellular positive allosteric modulator. Science 364, 1283-1287. doi: 10.1126/science.aaw8981

Livingston, K. E., Mahoney, J. P., Manglik, A., Sunahara, R. K., and Traynor, J. R. (2018). Measuring ligand efficacy at the mu-opioid receptor using a conformational biosensor. eLife, 7, e32499. doi.org/10.7554/eLife.32499

Masureel, M., Zou, Y., Picard, L. P., van der Westhuizen, E., Mahoney, J. P., Rodrigues, J., Mildorf, T. J., Dror, R. O., Shaw, D. E., Bouvier, M., Pardon, E., Steyaert, J., Sunahara, R. K., Weis, W. I., Zhang, C., and Kobilka, B. K. (2018). Structural insights into binding specificity, efficacy and bias of a β2AR partial agonist. Nat. Chem. Biol., 14, 1059–1066. doi.org/10.1038/s41589-018-0145-x

McMahon, C., Baier, A.S., Pascolutti, R., Wegrecki, M., Zheng, S., Ong, J.X., Erlandson, S.C., Hilger, D., Rasmussen, S.G.F., Ring, A.M., Manglik, A., and Kruse, A.C. (2018). Yeast surface display platform for rapid discovery of conformationally selective nanobodies. Nat. Struct. Mol. Biol. (3), 289-296. doi: 10.1038/s41594-018-0028-6

Nguyen, A.H., Thomsen, A.R.B., Cahill, T.J. 3rd, Huang, R., Huang, L.Y., Marcink, T., Clarke, O.B., Heissel, S., Masoudi, A., Ben-Hail, D., Samaan, F., Dandey, V.P., Tan, Y.Z., Hong, C., Mahoney, J.P., Triest, S., Little, J. 4th, Chen, X., Sunahara, R., Steyaert, J., Molina, H., Yu, Z., des Georges, A., and Lefkowitz, R.J. (2019). Structure of an endosomal signaling GPCR-G protein-β-arrestin megacomplex. Nat. Struct. Mol. Biol. 2019 26, 1123-1131. doi: 10.1038/s41594-019-0330-y

Rasmussen, S.G., Choi, H.J., Fung, J.J., Pardon, E., Casarosa, P., Chae, P.S., Devree, B.T., Rosenbaum, D.M., Thian, F.S., Kobilka, T.S., Schnapp, A., Konetzki, I., Sunahara, R.K., Gellman, S.H., Pautsch, A., Steyaert, J., Weis, W.I., and Kobilka, B.K. (2011a). Structure of a nanobody-stabilized active state of the β(2) adrenoceptor. Nature 469, 175-180. doi: 10.1038/nature09648

Rasmussen, S.G., DeVree, B.T., Zou, Y., Kruse, A.C., Chung, K.Y., Kobilka, T.S., Thian, F.S., Chae, P.S., Pardon, E., Calinski, D., Mathiesen, J.M., Shah, S.T., Lyons, J.A., Caffrey, M., Gellman, S.H., Steyaert, J., Skiniotis, G., Weis, W.I., Sunahara, R.K., and Kobilka, B.K (2011b). Crystal structure of the β2 adrenergic receptor-Gs protein complex. Nature 477, 549-555. doi: 10.1038/nature10361

Ring, A.M., Manglik, A., Kruse, A.C., Enos, M.D., Weis, W.I., Garcia, K.C., and Kobilka, B.K. (2013). Adrenaline-activated structure of β2-adrenoceptor stabilized by an engineered nanobody. Nature 502, 575-579. doi: 10.1038/nature12572

Sounier, R., Mas, C., Steyaert, J., Laeremans, T., Manglik, A., Huang, W., Kobilka, B. K., Déméné, H., & Granier, S. (2015). Propagation of conformational changes during μ-opioid receptor activation. Nature, 524, 375–378. doi.org/10.1038/nature14680

Staus, D. P., Wingler, L. M., Strachan, R. T., Rasmussen, S. G., Pardon, E., Ahn, S., Steyaert, J., Kobilka, B. K., and Lefkowitz, R. J. (2014). Regulation of β2-adrenergic receptor function by conformationally selective single-domain intrabodies. Mol. Pharmacol., 85, 472–481. [doi.org/10.1124/mol.113.089516](https://doi.org/10.1124/mol.113.089516)

Staus, D. P., Strachan, R. T., Manglik, A., Pani, B., Kahsai, A. W., Kim, T. H., Wingler, L. M., Ahn, S., Chatterjee, A., Masoudi, A., Kruse, A. C., Pardon, E., Steyaert, J., Weis, W. I., Prosser, R. S., Kobilka, B. K., Costa, T., and Lefkowitz, R. J. (2016). Allosteric nanobodies reveal the dynamic range and diverse mechanisms of G-protein-coupled receptor activation. Nature, 535, 448–452. doi.org/10.1038/nature18636

Stoeber, M., Jullié, D., Lobingier, B. T., Laeremans, T., Steyaert, J., Schiller, P. W., Manglik, A., and von Zastrow, M. (2018). A Genetically Encoded Biosensor Reveals Location Bias of Opioid Drug Action. Neuron, 98, 963–976.e5. doi.org/10.1016/j.neuron.2018.04.021

Vasudevan, L., and Stove, C.P. (2020). A novel nanobody-based bio-assay using functional complementation of a split nanoluciferase to monitor Mu- opioid receptor activation. Anal. Bioanal. Chem. 412, 8015-8022. doi: 10.1007/s00216-020-02945-6

Warne, T., Edwards, P. C., Doré, A. S., Leslie, A. G. W., and Tate, C. G. (2019). Molecular basis for high affinity agonist binding in GPCRs. Science 364, 775-778. doi.org/10.1126/science.aau5595

Westfield, G. H., Rasmussen, S. G., Su, M., Dutta, S., DeVree, B. T., Chung, K. Y., Calinski, D., Velez-Ruiz, G., Oleskie, A. N., Pardon, E., Chae, P. S., Liu, T., Li, S., Woods, V. L., Jr, Steyaert, J., Kobilka, B. K., Sunahara, R. K., and Skiniotis, G. (2011). Structural flexibility of the G alpha s alpha-helical domain in the beta2-adrenoceptor Gs complex. Proc. Natl. Acad. Sci. USA, 108, 16086–16091. doi.org/10.1073/pnas.1113645108

Wingler, L.M., McMahon, C., Staus, D.P., Lefkowitz, R.J., and Kruse, A.C. (2019). Distinctive Activation Mechanism for Angiotensin Receptor Revealed by a Synthetic Nanobody. Cell 176, 479-490. doi: 10.1016/j.cell.2018.12.006
